# Prevalence of antibiotic use for childhood diarrhoea in Uganda after an ORS scale-up intervention: a repeated cross-sectional study

**DOI:** 10.1186/s12889-024-19613-4

**Published:** 2024-08-01

**Authors:** Viktor Lundin, Felix Lam, Adewale Akinjeji, Lorraine Kabunga, Jaran Eriksen

**Affiliations:** 1 Department of Global Public Health, Karolinska Institutet, Stockholm, Sweden; 2 Clinton Health Access Initiative, Boston, USA; 3 ICAP Global Health Nigeria, Abuja, Nigeria; 4 Clinton Health Access Initiative, Kampala, Uganda; 5 Unit of infectious diseases/Venhälsan, Stockholm South Hospital, Stockholm, Sweden

**Keywords:** Diarrhoea, Antibiotics, Child Health, Uganda

## Abstract

**Background:**

Diarrhoea kills 500,000 children every year despite availability of cheap and effective treatment. In addition, a large number are inappropriately treated with antibiotics, which do not benefit the patient but can contribute to the development of antibiotic resistance. We investigated whether the prevalence of antibiotic use among children under the age of five with diarrhoea in Uganda changed following a national intervention to increase the use of oral rehydration salts (ORS), and whether any socioeconomic characteristics were associated with antibiotic use.

**Methods:**

A cross-sectional survey was conducted among caregivers of children under the age of five and among private health care providers and drug sellers in Uganda in 2014. This was compared to a similar survey among private health care providers, and the national demographic and health survey in Uganda in 2016. Logistic regression was used to find associations between antibiotic use and socioeconomic characteristics, and chi-square test and independent sample t-test were used to find significant differences between groups.

**Results:**

The prevalence of antibiotic use among children under the age of five with diarrhoea in Uganda decreased from 30.5% in 2014 to 20.0% (*p* < 0.001) in 2016. No associations between socioeconomic characteristics and the use of antibiotics were significant in both 2014 and 2016.

**Conclusions:**

The use of antibiotics in children with diarrhoeal disease decreased significantly in Uganda between 2014 and 2016. However, the extent of the contribution of the ORS scale-up programme to this decrease cannot be determined from this study.

**Supplementary Information:**

The online version contains supplementary material available at 10.1186/s12889-024-19613-4.

## Background

Globally, diarrhoeal disease causes approximately 500,000 childhood deaths annually [1, 2] of which most occur in south Asia and sub-Saharan Africa [1, 3]. In Uganda, the incidence of diarrhoeal disease has been estimated to 3.26 episodes per child per year, causing approximately 4500 deaths annually [1, 3]. Most of these deaths occur because of dehydration [4] which therefore is the main target of therapies when treating non-bloody diarrhoeal disease. The World Health Organization (WHO) recommends Oral Rehydration Salts (ORS) with zinc supplementation for most episodes of diarrhoea [5, 6] which is estimated to prevent up to 93% of diarrhoeal deaths [7]. Despite this, a large meta-analysis of data from 30 countries in sub-Saharan Africa found that antibiotics are still given to approximately 23% of children with non-bloody diarrhoea [8]. Another large study including children from South Africa and Tanzania, found that 21.5% and 48.5%, respectively, were given antibiotics for non-bloody diarrhoea [9].

Routine use of antibiotics for non-bloody diarrhoeal disease is actively discouraged by the WHO since it, due to non-bacterial aetiology of most diarrhoeal episodes, won’t benefit the patient [5] but might subject the patient to adverse effects [10]. Furthermore, overuse of antibiotics is known to be the main factor behind antibiotic resistance (ABR) [11], which is estimated to cause up to 10 million deaths globally by 2050 [12], and is considered one of the greatest threats to human health [13]. The widespread practice of non-prescribed consumption of antibiotics in low- and middle income countries (LMICs) put these settings at higher risk of developing ABR [14]. This, together with a greater burden of infectious diseases, and less developed health care systems suggest that the consequences of ABR will be the worst in poorer countries [15].

To reduce child mortality, several concrete interventions were launched by the Ugandan government and its partners to increase the use of ORS and zinc starting in 2010. For example, zinc was added to the national treatment guidelines for diarrhoea [16] and was allowed to be given as an over-the-counter medicine [17], and ORS and zinc began to be sold as a co-packaged product instead of being sold separately [17]. In 2010 the government also launched the integrated community case management program in which village health teams (VHTs) were trained to take care of common diseases at community level and to administer ORS and zinc to children with diarrhoea [17].

The Uganda Demographic and Health Survey in 2011 found that 45% of children under-5 with diarrhoea sought care from private sources [18]. To ensure patients seeking care in the private sector had access to ORS and zinc, health development partners to the Ministry of Health such as the Strengthening Health Outcomes through the Private Sector (SHOPS) project and the Clinton Health Access Initiative (CHAI) sought ways to improve availability of ORS and zinc and treatment practices among private health care providers and drug sellers. Between 2012 and 2016, health development partners implemented interventions such as training more than 12,000 private providers on diarrhoea management and hiring a team of ORS-promoters who visited more than 16,000 drug shops to educate on the seriousness of diarrhoeal disease and the benefits of ORS/zinc-treatment [17, 19]. In addition, campaigns in mass media, such as a national radio campaign targeting caregivers of children were launched with the aim to increase the knowledge of the benefits of ORS-treatment [17]. Details of these interventions are published elsewhere [17].

By 2016, the Demographic and Health Survey (DHS) in Uganda showed a significant increase in combined ORS and zinc coverage among children with a recent episode of diarrhoea, from 1% in 2011 to 30% in 2016 [17]. However, it is not known whether this intervention decreased the use of antibiotics for non-bloody diarrhoeal disease. This is information that could influence future policy and prove important to fight the future development of ABR.

We therefore wanted to assess and compare the prevalence of antibiotic use for treatment of diarrhoea in Ugandan children under five years of age, pre- and post the ORS scale-up programme. Our secondary aims were to compare the prevalence of antibiotic use for childhood diarrhoea between the urban and rural population, determine whether it differs depending on socioeconomic status of the caregiver or depending on whether care was sought at public or private health facilities.

## Methods

### Study design

To evaluate the change in the use of antibiotics, two cross-sectional surveys were conducted in Uganda in 2014 and 2016. A cross-sectional household survey consisting of interviews with caregivers of children under five was conducted by CHAI in 2014 (Supplement 1) and was compared to the 2016 DHS conducted by Uganda Bureau of Statistics (UBOS) (Uganda Demographic and Health Survey 2016^18^). Furthermore, cross-sectional surveys consisting of exit interviews with trained mock standardised patients visiting private medical outlets was conducted by CHAI in 2014 and 2016 (Supplement 2). Characteristics of the different surveys are described in Table 1.

**Table 1:** Overview of data sources

Survey name	Year	Target group	Conducted by	Sample size	Abbreviation
Mock standardised patient survey	2014	Private medical providers	CHAI	515	SP2014
Mock standardised patient survey	2016	Private medical providers	CHAI	688	SP2016
Household survey	2014	Caregivers of children under five	CHAI	5302	HH2014
Demographic health survey	2016	Caregivers of children under five	UBOS	15 522	DHS2016

### Household survey

For the surveys conducted by CHAI the enumerations areas (EAs) created for the 2002 population census in Uganda were used to select a representative randomized sample of the population. Prior to selection of EAs they were divided into regions that were characterized as urban or rural. Within each region, a sample of EAs were selected from the urban and rural group respectively with a ratio approximately equal to household proportion. In total, 169 EAs were randomly selected with probability proportional to size. In each of the selected EAs all households were mapped and the ones with at least one child under five were eligible for participation. From the list of eligible participants, households were randomly selected, and the survey was conducted, assessing treatment taken for childhood diarrhoea and the source of treatment. To be included in the study the person interviewed had to live within the selected EA, be the primary caregiver for a child under five, be over 18, and provide informed consent.

### Private medical outlet survey

Within each selected EA, community leaders and members were asked to list nearby medicine outlets and all private healthcare clinics that served community members. The health care clinics and medical outlets that provided consent were included in the study. Public health care clinics were not included. The private medical outlet survey was performed by a trained interviewer who sought out a mother with a child below five who was trained to remember a script describing acute, non-bloody diarrhoea in her child. She then visited a medical provider and asked for advice. Immediately afterwards the mother completed at short survey with the interviewer, regarding her interaction with the health care provider. The same survey questions were used in 2014 and 2016.

### Demographic and Health Survey

The DHS was performed by the Uganda Bureau of Statistics and is performed about every five years. For the DHS in 2016, a random sample of 696 EAs was selected and from each EA a representative number of households were randomly selected for participation. The resulting data were downloaded from UBOS website, and variables related to diarrhoea management for children who had an episode of diarrhoea in the last two weeks prior to the survey were extracted (Uganda Demographic and Health Survey 2016).

### Outcome measures

The primary outcome measures of the study were:

1. The proportion of children reported to have been given antibiotics for diarrhoea by their caregivers in 2014 compared to 2016 according to the CHAI household survey in 2014 and UBOS DHS in 2016.
2. The proportion of children that were prescribed antibiotics for diarrhoea by private medical providers in 2014 compared to 2016 according to the CHAI standardized patient survey.

The secondary outcome measures were the proportion of children that were given antibiotics depending on location (urban/rural), where they sought care (private/public health facilities), wealth quintile, sex, as well as depending on education level of the caregiver. Moreover, the proportion of children that were given antibiotics that also had fever, cough or difficulty breathing were analysed.

### Statistical analysis

Chi-square test was used to determine if the proportion of children that were prescribed antibiotics in 2014 and 2016 were significantly different. Means, standard deviations and frequencies were used to describe the characteristics such as age, sex, education level, place of residency, wealth, and frequency of diarrhoea as well as concomitant symptoms of the study participants. Furthermore, independent sample t-test was used to compare means between groups and logistic regression was used to the determine the level of association between different socioeconomic characteristics and the use of antibiotics. Level of significance was set to 0.05 and 95% confidence intervals for the found proportions were calculated using the Wald method. Statistical analysis was conducted using statistic software IBM SPSS Statistics 28.0 (SPSS Inc., Chicago, IL, USA).

## Results

### Demographic characteristics of the analysed surveys

A total of 5302 children under the age of five were recruited to the household survey in 2014 (HH2014) and 15,522 to the Demographic and Health survey in 2016 (DHS2016). The proportion of females were 51% (2706) in HH2014, and 49.5% (7,678) in DHS2016 (Table 1). The age of both children (2.27 **±** 1.4) and caregivers (32.5 ± 10.6) was higher in HH2014 compared to DHS2016 (1.97 **±** 1.4 and 28.5 ± 6.8). The majority of children lived in a rural area: 83.7% (4439) in HH2014 and 81.9 (12711) in DHS2016. The prevalence of children with diarrhoea in the two weeks prior to the survey was 24.9% (1318, 95% CI: 24–26) in HH2014 compared to 18.8% (2923, 95% CI: 18–19) in DHS2016. However, only 4.1% (215) in HH2014 and 4.3% (662) in DHS2016 had diarrhoea as the only symptom. The most common combination of symptoms was diarrhoea and fever which was found in 16.3% (865) of the children in HH2014 and 10.8% (1681) in DHS2016 (Table 2). Data on the frequency of bloody diarrhoea was not available for DHS2016 but in HH2014 the prevalence was 3.4% (178).

**Table 2:** Sociodemographic and illness characteristics of the caregivers and children under the age of five

	HH2014 (%, *n*) *n* = 5302		DHS2016 (%, *n*) *n* = 15 522	*p* ^a^
Sex of child				
Male	49.0 (2569)		50.5 (7844)	0.048
Female	51.0 (2706)		49.5 (7678)	0.048
Place of residency				
Urban	16.3 (863)		18.1 (2811)	0.003
Rural	83.7 (4439)		81.9 (12711)	0.003
Symptoms				
Diarrhoea in last 2 weeks	24.9 (1318)		18.8 (2923)	< 0.001
Bloody diarrhoea	3.4 (178)		N/A	N/A
Diarrhoea and fever	16.3 (865)		10.8 (1681)	< 0.001
Diarrhoea and cough	16.2 (859)		10.7 (1661)	< 0.001
Diarrhoea and difficulty breathing	6.7 (355)		5.8 (898)	0.016
Diarrhoea only	4.1 (215)		4.3 (662)	0.511
Mean age of child	2.27 **±** 1.4		1.97 **±** 1.4	< 0.001
Mean age of caregiver	32.5 ± 10.6		28.5 ± 6.8	< 0.001

Abbreviations: HH2014 – Household survey in 2014; DH20S16 – Demographic and Health Survey in 2016

^a^ Comparison of proportions. Chi-Square used for all except mean ages for which independent-sample t-test was used

### Prevalence of antibiotic use among children under five years with diarrhoea in Uganda

In 2014, 30.5% (402 of 1318, CI: 28–33) of the children with an episode of diarrhoea in the two weeks prior the survey were treated with antibiotics. This is significantly higher than the 20.0% (585 of 2923, CI: 19–21) that were treated antibiotics in 2016 (*p* < 0.001). The significantly lower proportion of antibiotic prescriptions for childhood diarrhoea in DHS2016 compared to HH2014 can, as shown in Table 3, be seen in almost all subgroups. The rate of reduction, however, was noticeably larger in some groups. In the urban population the use of antibiotics for childhood diarrhoea decreased from 36.4% (83 of 228, CI:30–43) to 17.7% (82 of 462, CI: 14–22, *p* < 0.001) and the prescription rate for children with a caregiver with no formal education decreased from 32.4% (68 of 210, CI: 26–39) in 2014 to 13.8% (50 of 363, CI: 10–17, *p* < 0.001) in 2016. Among the children raised in the wealthiest quintile prescription rates dropped from 35.2% (83 of 236, CI:29–41) to 17.2% (65 of 379, CI: 13–21, *p* < 0.001). Finally, children that sought care in the public health sector had a lower prevalence of antibiotic use in 2016 (138/890, 15.5%, CI: 13–18) compared to 2014 (145/521, 27.8%, CI: 24–32). No clear pattern could be found based on the education level of the caregiver.

**Table 3:** Prevalence of antibiotic use in children under five with diarrhoea

	HH2014		DHS2016		*p*
	Number of children	Prevalence (95% CI)	Number of children	Prevalence (95% CI)	
Total	402/1318	30.5 (28–33)	585 / 2923	20.0 (19–21)	< 0.001
Sex of child					
Male	202/656	30.8 (27–35)	301 / 1558	19.3 (17–21)	< 0.001
Female	200/662	30.2 (27–33)	284 / 1365	20.8 (19–23)	< 0.001
Place of residency					
Urban	83/228	36.4 (30–43)	82 / 462	17.7 (14–22)	< 0.001
Rural	319/1090	29.3 (27–32)	503 / 2,461	20.4 (18–22)	< 0.001
Care provider					
Private	164/530	30.9 (27–35)	344 / 1,151	29.9 (27–33)	0.661
Public	145/521	27.8 (24–32)	138 / 890	15.5 (13–18)	< 0.001
Wealth quintile					
Lowest	99 / 314	31.5 (26–37)	184 / 855	21.5 (19–24)	< 0.001
Second	79 / 258	30.6 (25–36)	133 / 668	19.9 (17–23)	< 0.001
Middle	60 / 249	24.1 (19–29)	112 / 552	20.3 (17–24)	0.225
Fourth	75 / 240	31.3 (25–37)	91 / 469	19.4 (16–23)	< 0.001
Highest	83 / 236	35.2 (29–41)	65 / 379	17.2 (13–21)	< 0.001
Concomitant symptoms					
Fever	269 / 865	31.1 (28–34)	380 / 1681	22.6 (21–25)	< 0.001
Cough	283 / 859	32.9 (30–36)	333 / 1661	20.0 (18–22)	< 0.001
Difficulty breathing	120 / 355	33.8 (29–39)	196 / 898	21.8 (19–25)	< 0.001
No other symptoms	58 / 215	27.0 (21–33)	112 / 662	16.9 (14–20)	0.001

*P*-values for the difference between HH2014 and DHS2016 were calculated through Chi-square test. Abbreviations: HH2014 – Household survey in 2014; DHS2016 – Demographic and Health Survey in 2016

### Socioeconomic factors in relationship to antibiotic use for childhood diarrhoea

Univariable analysis of the relationship between socioeconomic factors and the prescription of antibiotics for childhood diarrhoea found no associations that were statistically significant in both 2014 and 2016. The children in the rural population of HH2014 were less likely to be prescribed antibiotics for diarrhoeal disease (OR 0.72, 95% CI 0.56–0.98, *p* = 0.034) than their urban counterpart. In DHS2016, children with diarrhoeal disease that sought care in private health facilities were more than twice as likely to be prescribed antibiotics (OR 2.30, *p* < 0.001) than the ones that sought care at public health facilities. Furthermore, in 2016 children of caregivers with no education were less likely (OR 0.61, *p* = 0.003) than children of caregivers with primary education to receive antibiotics but no other significant association based on education was found.

Significant associations could also be seen between the use of antibiotics and other concomitant symptoms. The children in HH2014 were more likely to receive antibiotics if they also had cough (OR 1.4, *p* = 0.009) and in DHS2016 concomitant fever was significantly associated with the use of antibiotics (OR 1.47, *p* < 0.001). Likewise, the lack of other symptoms was also associated with less use of antibiotics (Table 4).

**Table 4:** Factors associated with use of antibiotics for childhood diarrhoea

	HH2014 OR (95% CI)	*P*-value	DHS2016 OR (95% CI)	*P*-value
Sex of child				
Male	1.00		1.00	
Female	0.97 (0.77–1.23)	0.819	1.01 (0.92–1.32)	0.316
Residency				
Urban	1.00		1.00	
Rural	**0.72 (0.56–0.98)**	**0.034**	1.19 (0.92–1.54)	0.185
Care provider				
Public	1.00		1.00	
Private	1.17 (0.91–1.51)	0.232	**2.30 (1.85–2.87)**	**< 0.001**
Wealth quintile				
Lowest	1.00		1.00	
Second	0.96 (0.67–1.37)	0.815	0.91 (0.71–1.16)	0.443
Middle	0.69 (0.47–1.0)	0.052	0.93 (0.71–1.21)	0.580
Fourth	0.99 (0.69–1.42)	0.944	0.88 (0.66–1.16)	0.878
Highest	1.18 (0.82–1.69)	0.369	0.76 (0.55–1.03)	0.755
Symptoms				
Fever	1.09 (0.85–1.40)	0.494	**1.47 (1.22–1.78)**	**< 0.001**
Cough	**1.40 (1.10–1.81)**	**0.009**	1.00 (0.83–1.20)	0.983
Difficulty breathing	1.23 (0.95–1.60)	0.121	1.17 (0.97–1.42)	0.107
Only diarrhoea	0.81 (0.59–1.13)	0.220	**0.78 (0.61–0.97)**	**0.024**

Abbreviations: HH2014 – Household survey in 2014; DHS2016 – Demographic and Health Survey in 2016; OR – Odds ratio

### Antibiotic prescription practices among private health care providers

After exclusion of 11 mock standardised patients in 2014 and 23 in 2016 that were revealed by the drug vendor, 504 private medical outlets in 2014 and 665 in 2016 were enrolled in the mock standardised patient survey (Table 5). The majority of the outlets consisted of drug shops: 64.9% (327, CI 61–69) in 2014 and 67.8% (451, CI 64–71) and the only significant differences that could be found between the two cohorts were the number of children under the age of 1 (23.0% in 2014, 18.0% in 2016, *p* = 0.036) and the distribution of medical outlets visited in the urban and rural sector respectively (*p* = 0.017).

**Table 5:** Sample characteristics in standardised patient survey among private medical outlets

	2014, % (*n*) *n* = 504	95% CI	2016, %, (*n*) *n* = 665	95% CI	*P*-value
Sex of child					
Male	50.2 (253)	46–55	52.3 (348)	49–56	0.470
Female	49.8 (251)	45–54	47.7 (317)	44–52	0.470
Residency					
Urban	30.8 (155)	27–35	37.4 (249)	34–41	0.017
Rural	69.2 (349)	65–73	62.6 (416)	59–66	0.017
Age of child					
<1	23.0 (116)	20–27	18.0 (120)	15–21	0.036
1–2	48.6 (245)	44–53	52.3 (348)	49–56	0.208
3–5	28.4 (143)	25–32	29.6 (197)	26–33	0.641
Outlet type					
Drug shop	64.9 (327)	61–69	67.8 (451)	64–71	0.292
Pharmacy	4.2 (21)	3–6	3.2 (21)	2–5	0.359
For-profit-clinic	28.4 (143)	25–32	28.9 (192)	26–32	0.852
Not-for-profit-clinic	2.6 (13)	2–4	0.2 (1)	0–0	-

In 2014, 46.4% (234 of 504, CI: 42–51) of the private medical outlets recommended antibiotics to the caregiver of a child under the age of five with watery diarrhoea compared to 47.7% (317 of 665, CI: 44–51) in 2016 (Table 6). The difference was not statistically significant (*p* = 0.674). Likewise, there was no significant difference between the urban and rural drug shops, nor depending on the sex of the child.

**Table 6:** Proportion of children with diarrhoea that were prescribed antibiotics in private medical outlets

	2014		2016		*p*
	Number of children	Prevalence (95% CI)	Number of children	Prevalence (95% CI)	
Total	234/504	46.4 (42–51)	317/665	47.7 (44–52)	0.674
Sex of child					
Male	114/253	45.1 (39–51)	170/348	48.9 (44–54)	0.358
Female	120/251	47.8 (42–54)	147/317	46.4 (41–52)	0.733
Residency					
Urban	63/155	40.6 (33–49)	120/249	48.2 (42–54)	0.138
Rural	171/349	49.0 (44–54)	197/416	47.4 (43–52)	0.651
Age of child					
<1	54/116	46.6 (38–56)	63/120	52.5 (44–61)	0.361
1–2	112/245	45.7 (40–52)	163/348	46.8 (42–52)	0.787
3–5	68/143	47.6 (40–56)	91/197	46.2 (39–53)	0.804
Outlet type					
Drug shop	163/327	49.8 (44–55)	214/451	47.5 (43–52)	0.509
Pharmacy	5/21	23.8 (10–45)	14/21	66.7 (45–84)	0.005
For-profit-clinic	61/143	42.7 (35–51)	89/192	46.4 (39–53)	0.501
Not-for-profit-clinic	5/13	38.5 (17–65)	0/1	-	-

Univariate analysis of the association between prescription rates for antibiotics and the different characteristics of the medical outlet showed no significant association between prescription of antibiotics and the location of the medical outlet, the sex, or the age of the child.

## Discussion

In this report, we used data gathered by CHAI and the DHS to get a more robust understanding of the development in the use of antibiotics in children under the age of five with diarrhoea in Uganda. Our findings suggest that the total prevalence of antibiotic use in children with diarrhoeal disease decreased significantly, from 30.5% in 2014 to 20.5% in 2016 and that the decrease was mainly driven by the public health sector. However, we found no significant associations between socioeconomic characteristics and the use of antibiotics that were consistent in both 2014 and 2016.

These results are somewhat surprising given that the global consumption of antibiotics among sick children in LMICs has been increasing steadily in the last decades [20–22]. This could be due to the increase being driven by treatment of respiratory symptoms rather than diarrhoea [20, 21]. Further support for our findings is found in the DHS reports of other sub-Saharan African countries where the use of antibiotics for diarrhoea seems to be decreasing [23–28]. It should also be noted that the antibiotic use for children with diarrhoea in both Uganda and Sub-Saharan Africa (23.1%) [8] is lower than the global average for LMICs (33.4%) [20, 21].

A decreasing use of antibiotics in contrast to the increasing global trend at the same time as availability and affordability of antibiotics has increased [29] suggests that interventions to change behaviour have had effect. However, because of our study design we are not able to attribute causality to any specific intervention. Moreover, interpretation of our results is further limited by the timing of the comparative surveys (only between 2014 and 2016). By 2014 some of the interventions intended to increase use of ORS were already implemented [17], and two years is a short period to follow such developments. On the other hand, the decrease in the use of antibiotics we found is supported by findings from the DHS in 2011 where the prevalence was almost the same as in 2014 (31.7%) [18].

Previous studies evaluating the consumption of antibiotics in Uganda have found a widespread overuse [30, 31], especially in hospitals, and that compliance with guidelines is low [32]. Although interpretation of possible overuse in our report is complicated by that most children with diarrhoea also had other concurrent symptoms, we found that 27% (HH2014) and 16.9% (DHS2016), respectively, received antibiotics despite having diarrhoea as the only symptom. Considering these known practices and the rising threat of ABR, the decreasing use of antibiotics in Uganda seem like a welcome development. However, although decreasing the use of antibiotics for diarrhoea might be an indicator of better antimicrobial stewardship, this is not necessarily the case. While viral causes were still the most common aetiology of childhood diarrhoea at the time of these surveys, bacterial infections seem to be more common than previous thought, and are likely to increase proportionally since the introduction of rotavirus in the immunization programmes [33–35]. One of the studies found that up to 37% of non-bloody diarrhoeal episodes were positive for *Shigella*,* Salmonella*,* Campylobacter*, ETEC or *Cryptosporidium*, and that bloody stools is a poor diagnostic and prognostic tool [34]. This casts doubt on the clinical relevance and effectivity of the WHO treatment algorithm. It is therefore important that future studies and interventions aiming to improve the use of antibiotics in LMICs focus on correct antibiotic targeting rather than just reduction.

The use of antibiotics for childhood diarrhoea was significantly reduced among children who were taken to public health facilities, while it remained practically unchanged in the private sector. Consequently, children with diarrhoea were more than twice as likely to receive antibiotics in a private health facility compared to a public in 2016. These findings were consistent with the standardized mock patient survey among private medical outlets where no significant change in antibiotic prescription was observed. Possible explanations for this are that it is easier to implement guidelines in public health facilities or that the profit incentives of private caregivers make them more likely to prescribe antibiotics. However, although there is support for an association between private caregivers and higher prescription rates in previous literature [8], there are also studies contradicting it [21, 32].

Moreover, the prescription rates of antibiotics in the standardized mock patient surveys were higher than those seen in HH2014 and DHS2016. This is likely because children that visit health facilities are more likely to use antibiotics [31], and not all children in the household surveys did. Furthermore, the ones that do seek care in a health facility are often the more severe cases, which might explain why studies analysing patients in health facilities generally find higher prescription rates (over 80%) than this report [30, 31].

The use of antibiotics for children with diarrhoea decreased more in the urban areas (from 36 to 17%) compared to the rural areas (29–20%). This development meant that the rural sector went from having significantly lower use of antibiotics in 2014 to not being significantly different from the urban sector in 2016. These findings are in line with previous studies which have suggested that the use of antibiotics in LMICs is higher in urban areas, although the relative increase in the use of antibiotics has been greater in the rural sector [8, 21]. A possible explanation for the larger decrease in urban areas could be a higher availability of better trained health professionals compared to the rural areas, which could make implementation of guidelines easier.

One limitation of the study is that the two community surveys (HH2014 and DHS2016) were not identical regarding the questionnaires used and how they were executed. Although HH2014 was designed to be similar to DHS2016 this is a source of uncertainty and complicates interpretation. Furthermore, the difference in the prevalence of diarrhoea of the surveys (24.9% in HH2014, 18.8% in DHS2016, *p* < 0.001) could imply that there are systematic differences between them. This discrepancy could partly be attributed to seasonal differences since the DHS2016 were conducted from June until December, and therefore partly during the dry months in the summer, while HH2014 was conducted in October – December and thereby mainly during the rainy season.

Moreover, interviewing caregivers regarding treatment and disease history of their children introduces recall bias, and both caregivers and interviewers might confuse which drugs are regarded as antibiotics or not. Another limitation is that this study only had two longitudinal data points with cross-sectional data. Adding more years to the study would have increased the reliability of it and would have made our findings on development of antibiotic prescription rates more robust. It should also be noted that we did numerous tests without changing the significance level. The data collection was completed almost eight years ago, and antibiotic use may very well have changed since then. However, our data do indicate that change in antibiotic use could happen fast, and what factors influence use, which is important for the field of antibiotic resistance. We therefore think this paper adds valuable data on the use of antibiotics in Uganda, which, in conjunction with the already existing literature, contributes to a better understanding of the situation.

## Conclusions

The prevalence of antibiotic use in children with diarrhoea in Uganda decreased significantly from 2014 to 2016. The decrease was greatest in urban settings and among children taken to public health facilities. However, because of the study design and the difference between the conducted surveys it cannot be determined whether this was because of the ORS-scale up or due to other reasons. Furthermore, multiple associations between socioeconomic characteristics of the caregiver and the use of antibiotics were found but none were consistent in both 2014 and 2016.

### Electronic supplementary material

Below is the link to the electronic supplementary material.

Supplementary Material 1

Supplementary Material 2

## Data Availability

The data for the DHS survey that support the findings of this study are available from the DHS programme, but restrictions apply to the availability of these data, which were used under license for the current study, and so are not publicly available. The datasets for HH2014 and the mock patient survey generated and/or analysed during the current study are not publicly available because they contain identifying patient information but are available from the corresponding author on reasonable request.

## Abbreviations

ABR: Antibiotic resistance
CHAI: Clinton Health Access Initiative
DHS: Demographic and health survey
DHS2016: Demographic and health survey in 2016
EA: Enumeration area
HH2014: Household survey 2014
LMIC: Low- and middle-income countries
ORS: Oral rehydration salts
SHOPS: Strengthening health outcomes through the private sector
UBOS: Uganda Bureau of Statistics
UN: United Nations
VHT: Village health teams
WHO: World Health Organization
