# Supplementary material for: Prevalence of antibiotic use for childhood diarrhoea in Uganda after an ORS scale-up intervention: a repeated cross-sectional study
Source: BMC Public Health. 2024 Aug 1;24:2084. doi: 10.1186/s12889-024-19613-4 (PMC11295441; doi:10.1186/s12889-024-19613-4)
Supplement: Supplementary file 1 — Supplementary Material 1 [file 12889_2024_19613_MOESM1_ESM.pdf]

SURVEY ID																

Household Questionnaire English Version

An assessment of the treatment of childhood illnesses in Uganda

SURVEY ID																

Table of Contents

MODULE A: COVER PAGE 3
MODULE B: HOUSEHOLD LISTING 5
MODULE C: ILLNESS DETAILS 7
MODULE D: KNOWLEDGE AND ATTITUDES 22
MODULE E: HOUSEHOLD CHARACTERISTICS 30
MODULE F: MESSAGE EXPOSURE 35
MODULE G: ENDING THE SURVEY 38

SURVEY ID | | | | | | | | | | | | | | | | |

MODULE A: COVER PAGE

Instructions: Complete **Section I: Identification** before you introduce yourself to the household caregiver

SECTION I: IDENTIFICATION			
**NO.**	**QUESTION**	**RESPONSE CODE**	**SKIP**
ID1	REGION CODE<br>*In ODK, select the name*	__ __	
ID2	DISTRICT CODE<br>*In ODK, select the name*	__ __ __	
ID3	COUNTY CODE<br>*In ODK, select the name*	__	
ID4	SUB-COUNTY CODE<br>*In ODK, select the name*	__ __	
ID5	PARISH CODE<br>*In ODK, select the name*	__ __	
ID6	VILLAGE CODE<br>*In ODK, select the name*	__ __	
ID7	ENUMERATION CODE<br>*In ODK, select the name*	__ __	
ID8	SECTOR (URBAN/RURAL)<br>*In ODK, select the only option available*	Urban .... 1<br>Rural 2	
ID9	HOUSEHOLD ID<br>*Obtain this information from the Household listing*	__ __	
ID10	SUPERVISOR CODE<br>*Select "Other" if you are a replacement interviewer*	__ __	
ID11	INVESTIGATOR CODE<br>*Select your name*	__ __ __ __	

SURVEY ID																

SECTION II: SCREENING QUESTIONS

NO.	QUESTION	RESPONSE CODE	SKIP
SQ1	HAVE YOU BEEN ABLE TO LOCATE THE HOUSEHOLD?	Yes .... 1<br>No 2	1 → SQ2<br>2 → MODULE G
SQ2	DOES THE HOUSEHOLD HAVE AT LEAST 1 CHILD UNDER AGE FIVE (0-59 MONTHS)?	Yes .... 1<br>No 2	1 → SQ3<br>2 → MODULE G
SQ3	IS THE RESPONDENT AGE 18 OR ABOVE?	Yes .... 1<br>No 2	1 → SQ4<br>2 → MODULE G
SQ4	HAVE YOU REVIEWED THE CONSENT FORM IN DETAIL WITH THE RESPONDENT AND HAS THE RESPONDENT PROVIDED CONSENT?	Yes .... 1<br>No 2	1 → HL1<br>2 → MODULE G

SURVEY ID

MODULE B: HOUSEHOLD LISTING

SECTION I: HOUSEHOLD LISTING

HL1	HOW MANY PEOPLE USUALLY SLEEP HERE?<br><br>*Write the number on line.*<br>*Special code:*<br>*Refused..97*<br>*Don't know....................................98*	Number of HH members .... ___ ___	
HL2	HOW MANY CHILDREN UNDER THE AGE OF FIVE (0-59 MONTHS) LIVE IN THIS HOUSEHOLD?<br><br>*Write the number on line.*<br>*Special code:*<br>*Refused..97*<br>*Don't know....................................98*	Number of children under 5.......... .... ___ ___	

INSTRUCTIONS FOR HL3-HL9 (ON NEXT PAGE):

Read to the respondent: PLEASE TELL ME THE NAME OF EACH CHILD UNDER FIVE WHO USUALLY LIVES AND SLEEPS IN THIS HOUSE.

List the names of each child under five in HL4 .Use a separate line for each child. After listing each child's name, ask questions HL5-HL9 for each child.

In the ODK system, put in the information about the first child by selecting 'Add group.' Upon completing all information about first child, then you will be asked to 'Add group' again for the second child. Continue to do this until you complete recording information about all children. Then select 'Do not add' in the group selection.

SURVEY ID | | | | | | | | | | | | | | | | |

HL3. LINE NO.	HL4. WHAT IS THE NAME OF THE CHILD? *Record the child's given name first and surname second. Record the child's initials if the respondent does not want to give the name.*	HL5. IS (*name*) MALE OR FEMALE? 1 Male 2 Female		HL6. HOW OLD IS (*name*)? *Record in completed years. E.g. If the child is less than 1 year, record `0'. If the child is 1 year and 8 months, record `1'. If the child is 1 year or greater, skip to HL8.*	HL7. IF THE CHILD IS LESS THAN 1 YEAR OLD, HOW OLD IS (*name*) **IN MONTHS**? *Only ask if HL6 is `0'. Record in completed months. E.g. If the child is 2 and a half months old, record `2'.*	HL8. HAS (NAME) HAD DIARRHOEA, FEVER, COUGH, OR FAST / DIFFICULTY BREATHING WITHIN THE LAST **TWO WEEKS**? *If "Yes", skip to Module C.*		HL9. HAS (NAME) HAD DIARRHOEA, FEVER, COUGH, OR FAST / DIFFICULTY BREATHING WITHIN THE LAST **FOUR WEEKS**? *Only ask if HL8 is `No'. If "Yes", go to Module C. If "No" return back to HL3 and add next child. In ODK, "Add group"*	
Line	Name	M	F	Age	Months	Y	N	Y	N
01		1	2	___	___ ___	1	2	1	2
02		1	2	___	___ ___	1	2	1	2
03		1	2	___	___ ___	1	2	1	2
04		1	2	___	___ ___	1	2	1	2
05		1	2	___	___ ___	1	2	1	2
06		1	2	___	___ ___	1	2	1	2
07		1	2	___	___ ___	1	2	1	2
08		1	2	___	___ ___	1	2	1	2
09		1	2	___	___ ___	1	2	1	2
10		1	2	___	___ ___	1	2	1	2

SURVEY ID | | | | | | | | | | | | | | | | |

MODULE C: ILLNESS DETAILS

SECTION I: ILLNESS DETAILS (DE)

Complete Module C for each child who had an illness in the last 2 or 4 weeks.

Read aloud to respondent: I WOULD NOW LIKE TO ASK YOU ABOUT (NAME'S) HEALTH, SPECIFICALLY ABOUT THE RECENT ILLNESS (NAME) HAS EXPERIENCED WITHIN THE LAST COUPLE OF WEEKS.

NO.	QUESTION	RESPONSE CODE	SKIP
DE1	DURING THE RECENT ILLNESS, DID (*name*) HAVE DIARRHOEA / STOOLING?	Yes .... 1<br>No .... 2<br>Refused .... 7<br>Don't know 8	1 → DE2<br>2 → DE5<br>7 → DE5<br>8 → DE5
DE2	DID (*NAME*) HAVE ANY BLOOD IN THE STOOLS WHEN HE OR SHE HAD DIARRHOEA / STOOLING?	Yes .... 1<br>No .... 2<br>Refused .... 7<br>Don't know 8	
DE3	FOR HOW MANY DAYS DID (NAME) HAVE DIARRHOEA / STOOLING?<br><br>*Write the number on line.*<br>*Special code:*<br>*Refused .... 97*<br>*Don't know 98*	Number of days .... ___ ___	
DE4	DOES (NAME) STILL HAVE DIARRHOEA / STOOLING?	Yes .... 1<br>No .... 2<br>Refused .... 7<br>Don't know 8	
DE5	DURING THE RECENT ILLNESS, DID (*name*) HAVE A FEVER?	Yes .... 1<br>No .... 2<br>Refused .... 7<br>Don't know 8	1 → DE6<br>2 → DE8<br>7 → DE8<br>8 → DE8
DE6	FOR HOW MANY DAYS DID (NAME) HAVE A FEVER?<br><br>*Write the number on line.*<br>*Special code:*<br>*Refused .... 97*<br>*Don't know 98*	Number of days .... ___ ___	

SURVEY ID | | | | | | | | | | | | | | | | |

DE7	DOES (NAME) STILL HAVE A FEVER?	Yes ....1<br>No.. ....2<br>Refused ....7<br>Don't know 8	
DE8	DURING THE RECENT ILLNESS, DID (*name*) HAVE A COUGH?	Yes ....1<br>No.. ....2<br>Refused ....7<br>Don't know 8	1 → DE9<br>2 → DE11<br>7 → DE11<br>8 → DE11
DE9	FOR HOW MANY DAYS DID (NAME) HAVE A COUGH?<br><br>*Write the number on line.*<br>*Special code:*<br>*Refused ....97*<br>*Don't know 98*	Number of days............................ ....___ ___	
DE10	DOES (NAME) STILL HAVE A COUGH?	Yes ....1<br>No.. ....2<br>Refused ....7<br>Don't know 8	
DE11	DURING THE RECENT ILLNESS, DID (*name*) HAVE FAST OR DIFFICULTY BREATHING?	Yes ....1<br>No.. ....2<br>Refused ....7<br>Don't know 8	1 → DE12<br>2 → DE15<br>7 → DE15<br>8 → DE15
DE12	WAS THE FAST OR DIFFICULTY BREATHING DUE TO A PROBLEM IN THE CHEST OR A BLOCKED OR RUNNY NOSE?	Problem in the chest only............... ....1<br>Blocked or runny nose only ....2<br>Both... ....3<br><br>Other (*specify*)________________ _________6<br>Refused ....7<br>Don't know 8	
DE13	FOR HOW MANY DAYS DID (NAME) HAVE FAST OR DIFFICULTY BREATHING?<br><br>*Write the number on line.*<br>*Special code:*<br>*Refused ....97*<br>*Don't know 98*	Number of days............................ ....___ ___	

SURVEY ID

DE14	DOES (NAME) STILL HAVE FAST OR DIFFICULTY BREATHING?	Yes .... 1<br>No .... 2<br>Refused .... 7<br>Don't know 8	
DE15	IN ADDITION, DID (NAME) HAVE ANY OF THE FOLLOWING SYMPTOMS:<br><br>*Prompt each response. If the respondent says "Yes" to a symptom, then select "Yes" as the answer. And if they say "No", then select "No". Use "Refused" and "Don't know" as applicable*	Yes / No / Ref / DK	
	[A] VOMITING?	A. Vomiting 1 2 7 8	
	[B] FATIGUE OR NO ENERGY?	B. Fatigue 1 2 7 8	
	[C] RESTLESS OR EASILY IRRITABLE?	C. Restless 1 2 7 8	
	[D] ABDOMINAL PAIN?	D. Abdominal pain 1 2 7 8	
	[E] PAIN ELSEWHERE?	E. Pain elsewhere 1 2 7 8	
	[F] THIRSTIER THAN USUAL?	F. Thirsty 1 2 7 8	
	[G] LESS URINE THAN NORMAL?	G. Decreased urine 1 2 7 8	
	[H] DARKER URINE THAN NORMAL?	H. Darker urine 1 2 7 8	
	[I] SUNKEN EYES?	I. Sunken eyes 1 2 7 8	
	[J] WRINKLY SKIN?	J. Wrinkly skin 1 2 7 8	
	[K] DRY OR STICKY MOUTH?	K. Dry or sticky mouth 1 2 7 8	
	[L] CONVULSIONS?	L. Convulsions 1 2 7 8	
	[M] COLD / SHIVERING?	M. Cold / shivering 1 2 7 8	
	[N] UNABLE TO EAT NORMALLY?	N. Unable to eat 1 2 7 8	
	[O] UNABLE TO DRINK NORMALLY?	O. Unable to drink 1 2 7 8	
	[P] UNABLE TO SLEEP NORMALLY?	P. Unable to sleep 1 2 7 8	
	[X] OTHER (*specify below*)?<br>[___________________________]	X. Other (specify) ___________________________	

SURVEY ID | | | | | | | | | | | | | | | | |

DE16	WHEN (*name*) FIRST SHOWED THESE SYMPTOMS, WHAT ILLNESS DID YOU THINK (*name*) HAD?	---	
DE17	HAS (*name*) HAD AN ILLNESS LIKE THIS BEFORE?	Yes ....1<br>No..2<br>Refused ....7<br>Don't know 8	1 → DE18<br>2 → DE19<br>7 → DE19<br>8 → DE19
DE18	COMPARED TO THE LAST TIME (*name*) HAD AN ILLNESS LIKE THIS, WOULD YOU SAY THE RECENT ILLNESS WAS:<br><br>LESS SEVERE, ABOUT THE SAME, OR MORE SEVERE THAN THE LAST TIME?	Somewhat less severe.................................1<br>About the same severe ....2<br>Somewhat more severe ....3<br>Refused ....7<br>Don't know 8	
DE19	DID YOU CONTINUE TO OFFER (*name*) FLUIDS TO DRINK OR BREASTMILK DURING THE ILLNESS?	Yes ....1<br>No..2<br>Refused ....7<br>Don't know 8	
DE20	DID YOU CONTINUE TO OFFER (*name*) FOOD TO EAT DURING THE ILLNESS?	Yes ....1<br>No..2<br>No, child is exclusively breastfed .. ..3<br>Refused ....7<br>Don't know 8	
DE21	DID YOU SEEK ADVICE OR TREATMENT FOR THE ILLNESS FROM ANY PLACE?	Yes ....1<br>No..2<br>Refused ....7<br>Don't know 8	1 → DE29<br>2 → DE22<br>7 → DE22<br>8 → DE22
DE22	WHY DID YOU NOT SEEK ANY ADVICE OR TREATMENT OUTSIDE OF YOUR HOME FOR THIS ILLNESS?<br><br>*Do not prompt. Mark all response mentioned as "Mentioned" and any responses not mentioned as "Not mentioned.*	The child got better without seeking care ....A<br>I did not think the illness needed outside care...B<br>I knew how to treat the illness at home ....C<br>The healthcare provider was too far.................D<br>I didn't have money to pay for medicines......... E<br>My family did not think the child needed to receive treatment outside the home ....F<br><br>Other (specify) _______________ ________ X<br><br>Refused ....Y<br>Don't know .... Z	

SURVEY ID

DE23	DID YOU GIVE ANY TREATMENT **FROM YOUR HOME**?	Yes .... 1<br>No.......... 2<br>Refused .... 7<br>Don't know 8	1 → DE24<br>2 → DE53<br>7 → DE53<br>8 → DE53
DE24	WHAT TREATMENTS DID YOU GIVE **FROM HOME**?<br><br>*Probe:*<br>ANYTHING ELSE?<br>*Ask to see the medicine if they still have it. Type the medicine exactly as indicated by respondent or labelled*	Medicine 1 ______ A<br>Medicine 2 ______ B<br>Medicine 3 ______ C<br>Medicine 4 ______ D<br>Medicine 5 ______ E	
DE25	CODE THE MEDICINE NAMES INTO CATEGORIES. ASK TO SEE THE MEDICINE PACKAGING IF IT IS AVAILABLE.<br><br>*Interviewers should use the show cards to categorize any medicine brands mentioned by the respondent into their appropriate categories. However, interviewers **should not** show the prompt cards to the respondent.*	Medicine 1 ______ A<br>Medicine 2 ______ B<br>Medicine 3 ______ C<br>Medicine 4 ______ D<br>Medicine 5 ______ E<br><br>Co-pack of ORS and Zinc .... 11<br>Oral rehydration salts (ORS).......... 12<br>Zinc (tablets or syrup) .... 13<br>Amoxicillin (tablets or syrup) .... 14<br>Cotrimoxazole (tablets or syrup).......... 15<br>Metronidazole (tablets or syrup) .... 16<br>Other antibiotics (tablets or syrup).......... 17<br>Anti-motility .... 18<br>Artemisinin combination therapy (ACTs).......... 19<br>Other antimalarials – not ACTs .... 20<br>Paracetamol.......... 21<br>IV fluids .... 22<br>Injection (antibiotic or non-antibiotic) .... 23<br>Home remedy (coconut water, juice, etc.).......... 24<br>Herbs.......... 25<br>Other .... 96<br>Refused .... 97<br>Don't know 98	
DE26	WHERE DID YOU OBTAIN THAT TREATMENT?<br><br>*Write the name of the place or of the person.*	--	

SURVEY ID | | | | | | | | | | | | | | | | |

DE27	IDENTIFY THE TYPE OF PROVIDER<br><br>*Probe to identify the type of source, but do NOT prompt with any suggestions*	Public sector<br>Govt. hospital ....11<br>Govt. HC II ....12<br>Govt. HC III ....13<br>Govt. HC IV ....14<br>Govt. Community Health Worker (CHW)...15<br>Private medical sector<br>Private hospital ....21<br>Private clinic ....22<br>Private doctor ....23<br>Pharmacy ....24<br>Drug shop ....25<br>Private Community Health Worker (CHW) 26<br>Other source<br>Relative / Friend ....31<br>Shop / Duka ....32<br>Traditional practitioner ....33<br>Market ....34<br>Home ....41<br><br>Other (*specify*)________________ ________96<br>Refused ....97<br>Don't know 98	
DE28	*If this place was included in the provider listing, write the provider code here.*<br><br>*If not listed, write 95*	Provider code ....___ ___	All responses → DE53
DE29	FROM HOW MANY PLACES DID YOU SEEK ADVICE OR TREATMENT?<br><br>*Write the number on line.*<br>*Special code:*<br>*Refused ....7*<br>*Don't know 8*	Number of places ....___	
DE30	BEFORE SEEKING ADVICE OR TREATMENT FROM ANY PLACE, DID YOU GIVE ANY TREATMENT THAT YOU ALREADY HAD **FROM YOUR HOME**?	Yes ....1<br>No ....2<br>Refused ....7<br>Don't know 8	1 → DE31<br>2 → DE36<br>7 → DE36<br>8 → DE36
DE31	WHAT TREATMENTS DO YOU GIVE **FROM HOME**?<br><br>*Probe:*<br>ANYTHING ELSE?<br>*Ask to see the medicine if they still have it. Type the medicine exactly as indicated by respondent or labelled*	Medicine 1 ____________________________ A<br><br>Medicine 2 ____________________________ B<br><br>Medicine 3 ____________________________ C<br><br>Medicine 4 ____________________________ D<br><br>Medicine 5 ____________________________ E	

SURVEY ID																

DE32	CODE THE MEDICINE NAMES INTO CATEGORIES. ASK TO SEE THE MEDICINE PACKAGING IF IT IS AVAILABLE.<br><br>*Interviewers should use the show cards to categorize any medicine brands mentioned by the respondent into their appropriate categories. However, interviewers **should not** show the prompt cards to the respondent.*	Medicine 1 ____________________________ A<br>Medicine 2 ____________________________ B<br>Medicine 3 ____________________________ C<br>Medicine 4 ____________________________ D<br>Medicine 5 ____________________________ E<br><br>Co-pack of ORS and Zinc .... 11<br>Oral rehydration salts (ORS)............................ 12<br>Zinc (tablets or syrup) .... 13<br>Amoxicillin (tablets or syrup) .... 14<br>Cotrimoxazole (tablets or syrup)...................... 15<br>Metronidazole (tablets or syrup) .... 16<br>Other antibiotics (tablets or syrup)................... 17<br>Anti-motility .... 18<br>Artemisinin combination therapy (ACTs)........ 19<br>Other antimalarials – not ACTs .... 20<br>Paracetamol.. 21<br>IV fluids .... 22<br>Injection (antibiotic or non-antibiotic) .... 23<br>Home remedy (coconut water, juice, etc.)........ 24<br>Herbs .... 25<br>Other .... 96<br>Refused .... 97<br>Don't know 98	
DE33	WHERE DID YOU OBTAIN THAT TREATMENT?<br><br>*Write the name of the place or of the person.*	--	

SURVEY ID

DE34	IDENTIFY THE TYPE OF PROVIDER<br><br>*Probe to identify the type of source, but do NOT prompt with any suggestions*	Public sector<br>Govt. hospital.......... 11<br>Govt. HC II.......... 12<br>Govt. HC III.......... 13<br>Govt. HC IV.......... 14<br>Govt. Community Health Worker (CHW)... 15<br>Private medical sector<br>Private hospital.......... 21<br>Private clinic.......... 22<br>Private doctor.......... 23<br>Pharmacy.......... 24<br>Drug shop.......... 25<br>Private Community Health Worker (CHW) 26<br>Other source<br>Relative / Friend.......... 31<br>Shop / Duka.......... 32<br>Traditional practitioner.......... 33<br>Market.......... 34<br>Home.......... 41<br><br>Other (*specify*)________ ________96<br>Refused.......... 97<br>Don't know.......... 98	
DE35	*If this place was included in the provider listing, write the provider code here.*<br><br>*If not listed, write 95*	Provider code.......... ___ ___	

SURVEY ID | | | | | | | | | | | | | | | | |

CARE-SEEKING LOCATIONS (DE36-DE44)

Instructions for DE36-DE44: *Please read the script below to the respondent.*

I WILL NOW ASK YOU ABOUT EACH PLACE WHERE YOU SOUGHT ADVICE OR TREATMENT. PLEASE TAKE YOUR TIME IN RESPONDING. THERE IS NO WRONG OR RIGHT ANSWER. FIRST, PLEASE TELL ME THE NAME OF THE FIRST PLACE WHERE YOU SOUGHT ADVICE OR TREATMENT.

In Line 01, complete DE37-DE44 for the first place where the respondent sought advice or treatment.

Then ask: DID YOU SEEK ADVICE OR TREATMENT FROM ANYONE ELSE?

If yes, complete questions DE37-DE44 for each place where the caregiver sought advice or treatment

In the ODK system, put in the information about the first Careseeking location by selecting 'Add group.' Upon completing all information about first location, then you will be asked to 'Add group' again for the second location. Continue to do this until you complete recording information about all locations. Then select 'Do not add' in the group selection.

DE36<br>LINE NO.	DE37<br>WHAT IS THE NAME OF THE PLACE WHERE YOU SOUGHT ADVICE OR TREATMENT?<br>*If the respondent does not know or remember the name of the place, write '98'.*	DE38<br>WHAT TYPE OF PLACE IS THIS?<br>*Code based on the description of the place. Do not prompt with suggestions or answer choices.*	DE39<br>DID YOU RECEIVE ANY MEDICINES FROM THIS PLACE?<br>*If "No", skip to DE44.*		DE40<br>WHAT ARE THE NAMES OF THE MEDICINES YOU RECEIVED?<br>*If available, ask for any leftover packaging. If the respondent does not know or remember the name of the medicine, write `Don't remember' for each medicine name that cannot be remembered.*		DE41<br>*Record the medicine code using the medicine catalogue.*	DE42<br>WHAT WAS THE TOTAL COST OF THE VISIT?<br>*If the cost is unknown, write `9998'.*	DE43<br>DID YOU REQUEST THESE MEDICINES SPECIFICALLY OR WERE THEY RECOMMENDED TO YOU BY THE TREATMENT SOURCE?			DE44<br>*If this place was included in the provider listing, write the provider code here. If the provider is not listed, write `95'.*
Line	Name	Type*	Y	N	No.	Medicine name	Medicine code	Total cost	Req	Rec	DK	Code
1		___ ___	1	2	[A]		___ ___		1	2	8	___ ___
					[B]		___ ___					
					[C]		___ ___					
					[D]		___ ___					
					[E]		___ ___					
2		___ ___	1	2	[A]		___ ___		1	2	8	___ ___
					[B]		___ ___					

SURVEY ID

				[C]		___ ___			
				[D]		___ ___			
				[E]		___ ___			
3		___ ___	1 2	[A]		___ ___		1 2 8	___ ___
				[B]		___ ___			
				[C]		___ ___			
				[D]		___ ___			
				[E]		___ ___			

*Codes for type of place where advice or treatment was sought

Public sector	Private sector	Other source
11 Government hospital	21 Private hospital	31 Relative / Friend
12 Government HC II	22 Private clinic	32 Shop / Duka
13 Government HC III	23 Private doctor	33 Traditional practitioner
14 Government HC IV	24 Pharmacy	34 Market
15 Government Community Health Worker (CHW)	25 Drug shop	96 Other
	26 Private Community Health Worker (CHW)	97 Refused
		98 Don't know

*Codes for medicines – select from the multiple choice options

11. Co-pack of ORS and Zinc	17. Other antibiotics (tablets or syrup)	23. Injection (antibiotic or non-antibiotic)
12. Oral rehydration salts (ORS)	18. Anti-motility	24. Home remedy (coconut water, juice, etc.)
13. Zinc (tablets or syrup)	19. Artemisinin combination therapy (ACTs)	25. Herbs
14. Amoxicillin (tablets or syrup)	20. Other antimalarials – not ACTs	96. Other (specify)
15. Cotrimoxazole (tablets or syrup)	21. Paracetamol	97. Refused
16. Metronidazole (tablets or syrup)	22. IV fluids	98. Don’t know

After DE44 in ODK:
Instructions: Ask the respondent if he or she went to any other place for advice or treatment. If “Yes”, swipe to the next screen and “Add group” of care-seeking location. If “No”, then select “Do not add” care-seeking location on the next screen.

SURVEY ID | | | | | | | | | | | | | | | | |

DE45	HOW MANY DAYS PASSED BETWEEN WHEN YOU FIRST NOTICED THE SYMPTOMS AND WHEN YOU SOUGHT ADVICE OR TREATMENT FROM THE FIRST SOURCE?<br><br>*Write the number on the line.*<br>*Special code:*<br>*Same day ....0*<br>*Refused ....97*<br>*Don't know 98*	Number of days ....___ ___	
DE46	WHAT SIGNS OR CONDITIONS OF (*name*) CAUSED YOU TO SEEK FOR CARE?<br><br>*Probe: ANYTHING ELSE?*<br><br>*Do not prompt. Mark all response mentioned as "Mentioned" and any responses not mentioned as "Not mentioned.*	Diarrhoea ....A<br>Fever ....B<br>Cough ....C<br>Fast breathing/difficulty to breath . ...D<br>Vomiting ....E<br>Fatigue or no energy .... F<br>Restlessness or easily irritable ....G<br>Pain ....H<br>Unconscious .... I<br>Convulsions ....J<br>Shivering or chills ....K<br>Unable to eat normally ....L<br>Unable to drink normally ....M<br>Unable to sleep normally ....N<br><br>Other (*specify*) _______________ _________ X<br><br>Refused ....Y<br>Don't know ....Z	
DE47	AT ANY POINT, DID YOU TELL THE PROVIDER OR MEDICINE SELLER WHAT ILLNESS **YOU** THOUGHT (*name*) HAD?	Yes.. .... 1<br>No .... 2<br>Refused .... 7<br>Don't know 8	
DE48	WAS (*name*) EXAMINED AT ANY OF THE PLACES YOU MENTIONED?	Yes.. .... 1<br>No .... 2<br>Refused .... 7<br>Don't know 8	1 → DE49<br>2 → DE53<br>7 → DE53<br>8 → DE53
DE49	AT ANY POINT DURING THE ILLNESS, DID (*name*) HAVE BLOOD TAKEN FROM HIS/HER FINGER OR HEEL FOR TESTING?	Yes.. .... 1<br>No .... 2<br>Refused .... 7<br>Don't know 8	

SURVEY ID

DE50	AT ANY POINT DURING THE ILLNESS, DID SOMEONE EXAMINE (*name's*) BREATHING, WITH:<br><br>[A] A STETHOSCOPE?<br><br>[B] A COUNTING DEVICE?<br><br>*PROMPT EACH RESPONSE.*<br>*Show stethoscope and respiratory rate counter prompt card.*	Yes No Ref DK<br>A. Stethoscope 1 2 7 8<br>B. Resp rate counter 1 2 7 8	
DE51	DID ANY OF THE SOURCES OF CARE PROVIDE A DIAGNOSIS OR EXPLAIN WHAT ILLNESS (NAME) HAD?	Yes.. .... 1<br>No.. .... 2<br>Refused.. .... 7<br>Don't know....................................... 8	1 → DE52<br>2 → DE53<br>7 → DE53<br>8 → DE53
DE52	WHAT WAS THE DIAGNOSIS GIVEN?	---	
DE53	AT ANY POINT DURING THE ILLNESS, WAS (*name*) OFFERED A FLUID MADE FROM A SPECIAL PACKET CALLED ORAL REHYDRATION SALT OR O.R.S?<br><br>*Show ORS prompt card*	Yes.. .... 1<br>No.. .... 2<br>Refused.. .... 7<br>Don't know....................................... 8	1 → DE54<br>2 → DE59<br>7 → DE59<br>8 → DE59
DE54	HOW MANY PACKETS OF ORS DID YOU PREPARE FOR (*name*) DURING THE ILLNESS?<br><br>*Write the number on line.*<br>*Special code:*<br>*Other (specify)............................96*<br>*Refused.......................................97*<br>*Don't know.................................98*	Number of ORS packets............... ....___ ___	
DE55	FOR HOW MANY DAYS DID YOU OFFER ORS TO (*name*)?<br><br>*Write the number on line.*<br>*Special code:*<br>*Until the diarrhoea stopped........95*<br>*Other (specify)............................96*<br>*Refused.......................................97*<br>*Don't know.................................98*	Number of days ....___ ___	

SURVEY ID | | | | | | | | | | | | | | | | |

DE56	HOW MANY TIMES IN A DAY DID YOU OFFER ORS TO (*name*)?<br><br>*Write the number on line.*<br>*Special code:*<br>*After each stool movement..........95*<br>*Other (specify)............................96*<br>*Refused.......................................97*<br>*Don't know.................................98*	Number of times a day.................. ....___ ___	
DE57	DID YOU DO ANYTHING TO TREAT THE WATER USED FOR MAKING ORS?	Yes.......... 1<br>No.......... 2<br>Refused.......... 7<br>Don't know.......... 8	1 → DE58<br>2 → DE59<br>7 → DE59<br>8 → DE59
DE58	WHAT DID YOU DO TO TREAT THE WATER?<br><br>*Probe:*<br>ANYTHING ELSE?<br><br>*Do not prompt. Mark all response mentioned as "Mentioned" and any responses not mentioned as "Not mentioned*	Boil..........A<br>Add bleach / chlorine..........B<br>Strain it through a cloth..........C<br>Use water filter (ceramic, sand, composite) ......D<br>Solar disinfection..........E<br>Let it stand and settle.......... F<br><br>Other (*specify*) _______________ _________ X<br><br>Refused..........Y<br>Don't know..........Z	
DE59	AT ANY POINT DURING THE ILLNESS, WAS (*name*) OFFERED ZINC TABLETS OR ZINC SYRUP?<br><br>*Show zinc prompt card.*	Yes.......... 1<br>No.......... 2<br>Refused.......... 7<br>Don't know.......... 8	1 → DE60<br>2 → DE63<br>7 → DE63<br>8 → DE63
DE60	FOR HOW MANY DAYS DID YOU OFFER ZINC TO (*name*)?<br><br>*Write the number on line.*<br>*Special code:*<br>*Until the diarrhoea stopped........95*<br>*Other (specify)............................96*<br>*Refused.......................................97*<br>*Don't know.................................98*	Number of days ....___ ___	

SURVEY ID | | | | | | | | | | | | | | | | |

DE61	HOW MANY TIMES IN A DAY DID YOU OFFER ZINC TO (*name*)?<br><br>*Write the number on line.*<br>*Special code:*<br>*After each stool movement..........95*<br>*Other (specify)............................96*<br>*Refused.......................................97*<br>*Don't know.................................98*	Number of times a day.................. ....___ ___	
DE62	HOW MUCH ZINC DID YOU OFFER TO (*name*) EACH TIME?	Half a tablet / half syrup cup.......... .... 1<br>1 tablet/ 1 syrup cup....................... .... 2<br>More than 1 tablet/ syrup cup .... 3<br><br>Other (*specify)* _______________ _________ 6<br>Refused.. .... 7<br>Don't know...................................... 8	
DE63	AT ANY POINT DURING THE ILLNESS, WAS (*name*) OFFERED AN ACT, SUCH AS COARTEM OR ARTEMETHER LUMEFANTRINE?<br><br>*Show ACT prompt card.*	Yes.. .... 1<br>No .... 2<br>Refused.. .... 7<br>Don't know...................................... 8	1 → DE64<br>2 → DE67<br>7 → DE67<br>8 → DE67
DE64	FOR HOW MANY DAYS DID YOU OFFER ACT TO (*name*)?<br><br>*Write the number on line.*<br>*Special code:*<br>*Until the illness stopped ....95*<br>*Other (specify)............................96*<br>*Refused.......................................97*<br>*Don't know.................................98*	Number of days ....___ ___	
DE65	HOW MANY TIMES IN A DAY DID YOU OFFER ACT TO (*name*)?<br><br>*Write the number on line.*<br>*Special code:*<br>*Other (specify)............................96*<br>*Refused.......................................97*<br>*Don't know.................................98*	Number of times a day.................. ....___ ___	

SURVEY ID | | | | | | | | | | | | | | | | |

DE66	HOW MUCH ACT DID YOU OFFER TO (*name*) EACH TIME?	Half a tablet .... 1<br>1 tablet .... 2<br>More than 1 tablet .... 3<br><br>Other (*specify*) ________________ 6<br>Refused .... 7<br>Don't know 8	
DE67	AT ANY POINT DURING THE ILLNESS, WAS (*name*) OFFERED AN ANTIBIOTIC (TABLET OR SYRUP)?<br><br>*Show antibiotic prompt card.*<br>*If "No", repeat Module C for each child with an illness in the last 2 or 4 weeks before moving on to KA1.*<br>*ODK Instructions: Are there any other children under five in the household? If "Yes", swipe to the next screen and "Add group". If "No", "Do not add" child group on the next screen.*	Yes .... 1<br>No .... 2<br>Refused .... 7<br>Don't know 8	1 → DE68<br>2 → KA1<br>7 → KA1<br>8 → KA1
DE68	FOR HOW MANY DAYS DID YOU GIVE THE ANTIBIOTIC TO (*name*)?<br><br>*Write the number on line.*<br>*Special code:*<br>*Until the illness stopped ....95*<br>*Other (specify)............................96*<br>*Refused.......................................97*<br>*Don't know................................98*	Number of days ....___ ___	
DE69	HOW FREQUENTLY DID YOU GIVE AN ANTIBIOTIC TO (*name*)?<br><br>*Write the number on line.*<br>*Special code:*<br>*Other (specify)............................96*<br>*Refused.......................................97*<br>*Don't know................................98*	Number of times a day..........................___ ___	
DE70	HOW MANY ANTIBIOTIC TABLETS OR SYRUP CUPS DID YOU GIVE TO (*name*) EACH TIME?<br><br>*Repeat Module C for each child with an illness in the last 2 or 4 weeks before moving on to KA1.*	Half a tablet / half a syrup cup....... .... 1<br>1 tablet / 1 syrup cup.................................... 2<br>More than 1 tablet / more than 1 syrup cup .... 3<br><br>Other (*specify*) ________________ 6<br>Refused .... 7<br>Don't know 8	
**After DE70 in ODK:**<br>**Instructions**: Ask the respondent "Are there any other children under five in the household?" If "Yes", swipe to the next screen and "Add group". If "No", "Do not add" child group on next screen.			

SURVEY ID | | | | | | | | | | | | | | | | |

MODULE D: KNOWLEDGE AND ATTITUDES

SECTION I: KNOWLEDGE AND ATTITUDES (KA)

NO.	QUESTION	RESPONSE CODE	SKIP
KA1	IF YOUR CHILD HAS DIARRHOEA, DO YOU PLAN TO SEEK CARE OR ADVICE OUTSIDE THE HOME?<br><br>*If the respondent says that the child has never had DIARRHOEA, create a hypothetical situation where the child does have diarrhoea, then ask respondent if he or she would seek care*	Yes .... 1<br>No .... 2<br>Refused .... 7<br>Don't know 8	
KA2	WHEN YOUR CHILD HAS DIARRHOEA, DO YOU KNOW WHAT TO GIVE TO YOUR CHILD AS TREATMENT?	Yes .... 1<br>No .... 2<br>Refused 7	1 → KA3<br>2 → KA6<br>7 → KA6
KA3	WHAT TREATMENTS DO YOU USUALLY GIVE YOUR CHILD IF HE OR SHE IS HAVING DIARRHOEA?<br><br>*Probe:*<br>ANYTHING ELSE?<br><br>*Use the medicines to directly code into categories. Ask to see the medicine packaging if it is available.*<br><br>*Mark all response mentioned as "Mentioned" and any responses not mentioned as "Not mentioned*	Co-pack of ORS and Zinc .... A<br>Oral rehydration salts (ORS) .... B<br>Zinc (tablets or syrup) .... C<br>Amoxicillin (tablets or syrup) .... D<br>Cotrimoxazole (tablets or syrup) .... E<br>Metronidazole (tablets or syrup) .... F<br>Other antibiotics (tablets or syrup) .... G<br>Anti-motility .... H<br>Artemisinin combination therapy (ACTs) .... I<br>Other antimalarials – not ACTs .... J<br>Paracetamol .... K<br>IV fluids .... L<br>Injection (antibiotic or non-antibiotic) .... M<br>Home remedy (coconut water, juice, etc.) .... N<br>Herbs .... O<br><br>Other (*specify*) ________________ X<br><br>Refused .... Y<br>Don't know .... Z	
KA4	DO YOU KNOW WHAT THE MINISTRY OF HEALTH (MOH) RECOMMENDS FOR TREATMENT OF CHILDREN'S DIARRHOEA?<br><br>*If answer is same as listing of medicines before, select SAME MEDICINES AS BEFORE. Otherwise, click YES and select the medicine category as indicated in next question.*	Yes .... 1<br>No .... 2<br>Same medicines as before .... 3<br>Refused 7	1 → KA5<br>2 → KA6<br>3 → KA6<br>7 → KA6

SURVEY ID | | | | | | | | | | | | | | | | |

KA5	WHAT IS THE MOH RECOMMENDED TREATMENT FOR CHILDREN'S DIARRHOEA?<br><br>*Probe:*<br>ANYTHING ELSE?<br><br>*Use the medicines to directly code into categories. Ask to see the medicine packaging if it is available.*<br><br>*Mark all response mentioned as "Mentioned" and any responses not mentioned as "Not mentioned*	Co-pack of ORS and Zinc .... A<br>Oral rehydration salts (ORS)............................ B<br>Zinc (tablets or syrup)...................................... C<br>Amoxicillin (tablets or syrup).......................... D<br>Cotrimoxazole (tablets or syrup)........................E<br>Metronidazole (tablets or syrup)........................F<br>Other antibiotics (tablets or syrup).................... G<br>Anti-motility .... H<br>Artemisinin combination therapy (ACTs) ....I<br>Other antimalarials – not ACTs .... J<br>Paracetamol... K<br>IV fluids ....L<br>Injection (antibiotic or non-antibiotic)............. M<br>Home remedy (coconut water, juice, etc.) .... N<br>Herbs.. O<br><br>Other (*specify*) _________________________ X<br><br>Refused .... Y<br>Don't know ....Z	
KA6	BEFORE TODAY, HAVE YOU HEARD OF A SPECIAL PRODUCT CALLED ORAL REHYDRATION SALTS (ORS) THAT YOU CAN GET FOR THE TREATMENT OF DIARRHOEA?<br><br>*Show respondent ORS prompt card.*	Yes .... 1<br>No .... 2<br>Refused .... 7<br>Don't know 8	1 → KA7<br>2 → KA11<br>7 → KA11<br>8 → KA11
KA7	DO YOU KNOW HOW TO GIVE ORS TO YOUR CHILD IF HE/SHE HAS DIARRHOEA?	Yes .... 1<br>No .... 2<br>Refused 7	1 → KA8<br>2 → KA10<br>7 → KA10
KA8	FOR HOW LONG SHOULD ORS BE GIVEN TO YOUR CHILD IF HE/SHE HAS DIARRHOEA?<br><br>*Write the number on line.*<br>*Special code:*<br>*Until the diarrhoea ends ....95*<br>*Other (specify)............................96*<br>*Refused.......................................97*<br>*Don't know.................................98*	Number of days.................................... ___ ___	

SURVEY ID | | | | | | | | | | | | | | | | |

KA9	HOW FREQUENTLY SHOULD ORS BE GIVEN TO YOUR CHILD IF HE/SHE HAS DIARRHOEA?<br><br>*Write the number on line.*<br>*Special code:*<br>*After each stool movement. ....95*<br>*Other (specify)............................96*<br>*Refused.......................................97*<br>*Don't know.................................98*	Number of time per day .... ___ ___	
KA10	IN YOUR OPINION, WHAT IS THE BENEFIT OF ORS?<br><br>*Do not prompt. Mark all response mentioned as "Mentioned" and any responses not mentioned as "Not mentioned*	Prevents dehydration ....A<br>Rehydrates a dehydrated child...........................B<br>Prevents future diarrhoea...................................C<br>Helps child recover quickly from diarrhoea ......D<br>Slows or stops diarrhoea....................................E<br>Gives child strength or energy after they have had diarrhoea..F<br>Strengthens immunity..G<br><br>Other (*specify*) _________________________X<br><br>Refused..Y<br>Don't know..Z	
KA11	BEFORE TODAY, HAVE YOU HEARD OF A SPECIAL MEDICINE CALLED ZINC YOU CAN GET FOR THE TREATMENT OF DIARRHOEA?<br><br>*Show respondent zinc prompt card.*	Yes ....1<br>No ....2<br>Refused ....7<br>Don't know 8	1 → KA12<br>2 → KA17<br>7 → KA17<br>8 → KA17
KA12	DO YOU KNOW HOW TO GIVE ZINC TO YOUR CHILD IF HE/SHE HAS DIARRHOEA?	Yes ....1<br>No ....2<br>Refused 7	1 → KA13<br>2 → KA16<br>7 → KA16
KA13	FOR HOW MANY DAYS SHOULD ZINC BE GIVEN TO YOUR CHILD IF HE/SHE HAS DIARRHOEA?<br><br>*Write the number on line.*<br>*Special code:*<br>*Until the diarrhoea ends ....95*<br>*Other (specify)............................96*<br>*Refused.......................................97*<br>*Don't know.................................98*	Number of days............................ .... ___ ___	

SURVEY ID | | | | | | | | | | | | | | | | |

KA14	HOW FREQUENTLY SHOULD YOU GIVE ZINC TO (*name*)?<br><br>*Write the number on line.*<br>*Special code:*<br>*After each stool movement. ....95*<br>*Other (specify)............................96*<br>*Refused.......................................97*<br>*Don't know.................................98*	Number of times a day................. .... ___ ___	
KA15	HOW MANY ZINC TABLETS OR SYRUP CUPS SHOULD YOU GIVE TO (*name*) EACH TIME?	Half a tablet / half syrup cup.......... ....1<br>1 tablet/ 1 syrup cup...................... ....2<br>More than 1 tablet/ syrup cup ....3<br><br>Other (*specify)* _______________ _________ 6<br>Refused ....7<br>Don't know 8	
KA16	IN YOUR OPINION, WHAT IS THE BENEFIT OF ZINC?<br><br>*Do not prompt. Mark all response mentioned as "Mentioned" and any responses not mentioned as "Not mentioned*	Prevents dehydration ....A<br>Rehydrates a dehydrated child...........................B<br>Prevents future diarrhoea..................................C<br>Helps child recover quickly from diarrhoea ......D<br>Slows or stops diarrhoea...................................E<br>Gives child strength or energy after they have had diarrhoea..F<br>Strengthens immunity.......................................G<br><br>Other (*specify)* _________________________X<br><br>Refused..Y<br>Don't know..Z	
KA17	BEFORE TODAY, HAVE YOU EVER HEARD OF AN ILLNESS CALLED PNEUMONIA?	Yes ....1<br>No ....2<br>Refused ....7<br>Don't know 8	1 → KA18<br>2 → KA21<br>7 → KA21<br>8 → KA21

SURVEY ID

KA18	WHAT SYMPTOMS DOES A CHILD USUALLY HAVE IF HE OR SHE HAS PNEUMONIA?<br><br>*Probe:*<br>ANYTHING ELSE?<br><br>*Do not prompt. Record all items mentioned. Mark all response mentioned as "Mentioned" and any responses not mentioned as "Not mentioned*	Diarrhoea .... A<br>Fever .... B<br>Cough .... C<br>Fast breathing/difficulty to breath .... D<br>Vomiting .... E<br>Fatigue or no energy .... F<br>Restlessness or easily irritable .... G<br>Pain .... H<br>Unconscious .... I<br>Convulsions .... J<br>Shivering or chills .... K<br>Unable to eat normally .... L<br>Unable to drink normally .... M<br>Unable to sleep normally .... N<br><br>Other (*specify*) ________ X<br><br>Refused .... Y<br>Don't know .... Z	
KA19	DO YOU KNOW WHAT TO GIVE YOUR CHILD IF HE OR SHE HAS PNEUMONIA?	Yes .... 1<br>No .... 2<br>Refused 7	1 → KA20<br>2 → KA21<br>7 → KA21
KA20	WHAT TREATMENTS DO YOU USUALLY GIVE YOUR CHILD IF HE OR SHE IS HAVING SYMPTOMS SHOWING POSSIBLE PNEUMONIA?<br><br>*Probe:*<br>ANYTHING ELSE?<br><br>*Use the medicines to directly code into categories. Ask to see the medicine packaging if it is available.*<br><br>*Mark all response mentioned as "Mentioned" and any responses not mentioned as "Not mentioned*	Co-pack of ORS and Zinc .... A<br>Oral rehydration salts (ORS) .... B<br>Zinc (tablets or syrup) .... C<br>Amoxicillin (tablets or syrup) .... D<br>Cotrimoxazole (tablets or syrup) .... E<br>Metronidazole (tablets or syrup) .... F<br>Other antibiotics (tablets or syrup) .... G<br>Anti-motility .... H<br>Artemisinin combination therapy (ACTs) .... I<br>Other antimalarials – not ACTs .... J<br>Paracetamol .... K<br>IV fluids .... L<br>Injection (antibiotic or non-antibiotic) .... M<br>Home remedy (coconut water, juice, etc.) .... N<br>Herbs .... O<br><br>Other (*specify*) ________ X<br><br>Refused .... Y<br>Don't know .... Z	
KA21	BEFORE TODAY, HAVE YOU EVER HEARD OF AN ILLNESS CALLED MALARIA?	Yes .... 1<br>No .... 2<br>Refused .... 7<br>Don't know 8	1 → KA22<br>2 → KA29<br>7 → KA29<br>8 → KA29

SURVEY ID | | | | | | | | | | | | | | | | |

KA22	WHAT SYMPTOMS DOES A CHILD USUALLY HAVE IF HE OR SHE HAS MALARIA?<br><br>*Probe:*<br>ANYTHING ELSE?<br><br>*Do not prompt. Mark all response mentioned as "Mentioned" and any responses not mentioned as "Not mentioned*	Diarrhoea .... A<br>Fever .... B<br>Cough .... C<br>Fast breathing/difficulty to breath.. .... D<br>Vomiting .... E<br>Fatigue or no energy .... F<br>Restlessness or easily irritable .... G<br>Pain .... H<br>Unconscious .... I<br>Convulsions .... J<br>Shivering or chills .... K<br>Unable to eat normally .... L<br>Unable to drink normally .... M<br>Unable to sleep normally .... N<br><br>Other (*specify*) ________________ __________ X<br><br>Refused .... Y<br>Don't know .... Z	
KA23	DO YOU KNOW WHAT TO GIVE YOUR CHILD IF HE OR SHE HAS MALARIA?	Yes .... 1<br>No .... 2<br>Refused 7	1 → KA24<br>2 → KA27<br>7 → KA27
KA24	WHAT TREATMENTS DO YOU USUALLY GIVE YOUR CHILD IF HE OR SHE HAS MALARIA?<br><br>*Probe:*<br>ANYTHING ELSE?<br><br>*Use the medicines to directly code into categories. Ask to see the medicine packaging if it is available.*<br><br>*Mark all response mentioned as "Mentioned" and any responses not mentioned as "Not mentioned*	Co-pack of ORS and Zinc .... A<br>Oral rehydration salts (ORS) .... B<br>Zinc (tablets or syrup) .... C<br>Amoxicillin (tablets or syrup) .... D<br>Cotrimoxazole (tablets or syrup) .... E<br>Metronidazole (tablets or syrup) .... F<br>Other antibiotics (tablets or syrup) .... G<br>Anti-motility .... H<br>Artemisinin combination therapy (ACTs) .... I<br>Other antimalarials – not ACTs .... J<br>Paracetamol .... K<br>IV fluids .... L<br>Injection (antibiotic or non-antibiotic) .... M<br>Home remedy (coconut water, juice, etc.) .... N<br>Herbs .... O<br><br>Other (*specify*) __________________________ X<br><br>Refused .... Y<br>Don't know .... Z	

SURVEY ID

KA25	DO YOU KNOW WHAT THE MINISTRY OF HEALTH (MOH) RECOMMENDS FOR TREATMENT OF MALARIA IN CHILDREN?<br><br>*If answer is same as listing of medicines before, select SAME MEDICINES AS BEFORE. Otherwise, click YES and select the category of medicines as indicated in next question.*	Yes .... 1<br>No .... 2<br>Same medicines as before .... 3<br>Refused 7	1 → KA26<br>2 → KA27<br>3 → KA27<br>7 → KA27
KA26	WHAT IS THE MOH RECOMMENDED TREATMENT FOR MALARIA IN CHILDREN?<br><br>*Probe:*<br>ANYTHING ELSE?<br><br>*Use the medicines to directly code into categories. Ask to see the medicine packaging if it is available.*<br><br>*Mark all response mentioned as "Mentioned" and any responses not mentioned as "Not mentioned*	Co-pack of ORS and Zinc .... A<br>Oral rehydration salts (ORS) .... B<br>Zinc (tablets or syrup) .... C<br>Amoxicillin (tablets or syrup) .... D<br>Cotrimoxazole (tablets or syrup) .... E<br>Metronidazole (tablets or syrup) .... F<br>Other antibiotics (tablets or syrup) .... G<br>Anti-motility .... H<br>Artemisinin combination therapy (ACTs) .... I<br>Other antimalarials – not ACTs .... J<br>Paracetamol .... K<br>IV fluids .... L<br>Injection (antibiotic or non-antibiotic) .... M<br>Home remedy (coconut water, juice, etc.) .... N<br>Herbs .... O<br><br>Other (*specify*) ________________ X<br><br>Refused .... Y<br>Don't know .... Z	
KA27	BEFORE TODAY, HAVE YOU EVER HEARD OF A RAPID DIAGNOSTIC TEST, OR RDT, FOR DIAGNOSING MALARIA?<br><br>*Show RDT prompt card.*	Yes .... 1<br>No .... 2<br>Refused .... 7<br>Don't know 8	
KA28	BEFORE TODAY, HAVE YOU EVER HEARD OF ARTEMISININ COMBINATION TREATMENT, OR ACT, FOR TREATING MALARIA?<br><br>*Show ACT prompt card.*	Yes .... 1<br>No .... 2<br>Refused .... 7<br>Don't know 8	

SURVEY ID | | | | | | | | | | | | | | | | |

CARESEEKING LOCATIONS (KA29-KA36)

Instructions for KA29-KA36: *Please read the script below to the respondent.*

I WOULD LIKE TO KNOW ABOUT ALL THE PLACES WHERE YOU SEEK ADVICE OR TREATMENT WHEN YOUR CHILD IS ILL. CAN YOU PLEASE TELL ME ALL THE PLACES WHERE YOU SEEK ADVICE OR TREATMENT WHEN YOUR CHILD IS ILL?

In the ODK system, put in the information about the first Careseeking location by selecting 'Add group.' Upon completing all information about first location, then you will be asked to 'Add group' again for the second location. Continue to do this until you complete recording information about all locations. Then select 'Do not add' in the group selection.

KA29 LINE NO.	KA30 WHAT ARE THE NAMES OF THE PLACE WHERE YOU GO TO FOR ADVICE OR TREATMENT? *If the respondent does not know or remember the name of the place, write '98'.*	KA31 WHAT IS THE TYPE OF PLACE YOU GO TO FOR ADVICE OR TREATMENT?	KA32 HOW SATISFIED ARE YOU WITH THE QUALITY OF CARE AT THIS PLACE? VERY GOOD, AVERAGE, POOR?				KA33 WHEN YOU GO TO THIS FACILITY, HOW OFTEN DO THEY HAVE MEDICINES? ALWAYS, SOMETIMES, OR NEVER?				KA34 HOW AFFORDABLE ARE THE MEDICINES AND SERVICES AT THIS PLACE ARE? FREE MEDICINES AND SERVICES, AFFORDABLE, OR EXPENSIVE?				KA35 WHEN SEEKING TREATMENT FROM THIS PLACE, DO YOU NORMALLY REQUEST A SPECIFIC MEDICINE OR ASK FOR A RECOMMENDATION?			KA36 *If this place was included in the provider listing, write the provider code here Write 95 if not listed.*
Line	Name	Type*	VG	A	P	DK	A	S	N	DK	F	A	E	DK	Req	Rec	DK	Code
1		___ ___	1	2	3	**8**	1	2	3	8	1	2	3	8	1	2	8	___ ___
2		___ ___	1	2	3	8	1	2	3	8	1	2	3	8	1	2	8	___ ___
3		___ ___	1	2	3	8	1	2	3	8	1	2	3	8	1	2	8	___ ___
4		___ ___	1	2	3	8	1	2	3	8	1	2	3	8	1	2	8	___ ___
5		___ ___	1	2	3	8	1	2	3	8	1	2	3	8	1	2	8	___ ___

*Codes for type of place respondent goes to for advice or treatment

Public sector	Private sector	Other source
11 Government hospital	21 Private hospital	31 Relative / Friend
12 Government HC II	22 Private clinic	32 Shop / Duka
13 Government HC III	23 Private doctor	33 Traditional practitioner
14 Government HC IV	24 Pharmacy	34 Market
15 Government Community Health Worker (CHW)	25 Drug shop	96 Other
	26 Private Community Health Worker (CHW)	97 Refused
		98 Don't know

After KA36 in ODK:
Instructions: Ask the respondent if he or she goes to any other place for advice or treatment. If "Yes", swipe to the next screen and "Add group" of care-seeking location. If "No", then select "Do not add" care-seeking location.

SURVEY ID

MODULE E: HOUSEHOLD CHARACTERISTICS

SECTION I: RESPONDENT DETAILS

NO.	QUESTION	RESPONSE CODE	SKIP
RD1	WHAT IS YOUR AGE?<br><br>*Write the number on line.*<br>*Answer should be between 18 and 98*<br>*Special code:*<br>*95 or older.................................95*<br>*Refused......................................97*<br>*Don't know................................98*	Age of respondent........................ .... ___ ___	
RD2	*Is the respondent male or female?*	Male.. .... 1<br>Female 2	
RD3	WHAT IS THE HIGHEST LEVEL OF SCHOOL YOU ATTENDED: PRIMARY, `O' LEVEL, `A' LEVEL, OR UNIVERSITY OR TERTIARY?	Primary .... 11<br>`O' level........................................ ................. 12<br>`A'level.. .... 13<br>Tertiary .... 14<br>University .... 15<br>Never attended school .... 16<br>Refused .... 97<br>Don't know.................................... 98	
RD4	WHAT IS YOUR RELIGION?	Catholic.. .... 1<br>Protestant .... 2<br>Muslim.. .... 3<br>Pentecostal .... 4<br>SDA .... 5<br><br>Other (*specify*) _______________ _________ 6<br>Refused .... 7<br>Don't know.................................... 8	
RD5	WHAT IS YOUR TRIBE?	Muganda .... 1<br>Munyankole .... 2<br>Musoga .... 3<br>Mukiga.. .... 4<br>Ateso... .... 5<br><br>Other (*specify*) _______________ _________ 6<br>Refused .... 7<br>Don't know.................................... 8	

SURVEY ID

RD6	WHAT IS YOUR RELATIONSHIP TO THE HEAD OF HOUSEHOLD?	Head of household .... 11<br>Wife / Husband.............................. .... 12<br>Son / Daughter .... 13<br>Son-In-Law / Daughter-In-Law .... 14<br>Grandchild .... 15<br>Parent... .... 16<br>Parent-In-Law................................. .... 17<br>Brother / Sister............................... .... 18<br>Brother-In-Law / Sister-In-Law..... .... 19<br>Uncle / Aunt................................... .... 20<br>Niece / Nephew .... 21<br>Other relative .... 22<br>Adopted / Foster / Stepchild .... 23<br>Not related .... 24<br><br>Other (specify)_______________ ________ 96<br>Refused .... 97<br>Don't know..................................... 98	1 → HC1
RD7	WHAT IS THE AGE OF THE HEAD OF HOUSEHOLD?<br><br>*Write the number on line.*<br>*Special code:*<br>*95 or older.................................95*<br>*Refused......................................97*<br>*Don't know.................................98*	Age of HH head .... ___ ___	

**SECTION II: HOUSEHOLD CHARACTERISTICS**			
HC1	*Main material of the dwelling floor.*<br><br>*Record observation.*<br>*Do not ask respondent unless you are interviewing outside of the respondent's home. If you do ask, do so in a polite manner.*	Natural floor (Earth / Sand / Dung) .... 10<br>Rudimentary floor (Wood planks/Bamboo) ... 20<br>Finished floor (Cement / Polished wood / Tiles / Bricks / Stones) .... 30<br><br>Other (*specify*) _______________ ________ 96	
HC2	*Main material of the roof.*<br><br>*Record observation.*<br>*Do not ask respondent unless you are interviewing outside of the respondent's home. If you do ask, do so in a polite manner.*	Natural roofing (Thatch/Mud/Palm leaf)......... 10<br>Rudimentary Roofing (Palm fronds/ Bamboo/ Wood planks / Plastic / Cardboard / Polythene) .... 20<br>Finished roofing (Iron sheets / Tiles / Tin / Cement / Asbestos)................................ .... 30<br><br>Other (*specify*) _______________ ________ 96	

SURVEY ID | | | | | | | | | | | | | | | | |

HC3	*Main material of the exterior walls.*<br><br>*Record observation.*<br>*Do not ask respondent unless you are interviewing outside of the respondent's home. If you do ask, do so in a polite manner.*	Natural walls (Thatched / Straw) ... . 10<br>Rudimentary walls (Mud and poles / un-burnet bricks / burnt bricks with mud) .... 20<br>Finished walls (Cement / Stone / Timber) .... 30<br><br>Other (*specify*) _______________ ________ 96	

			Yes	No	Ref	DK	
HC4	DOES YOUR HOUSEHOLD HAVE:						
	[A] ELECTRICITY?	A. Electricity	1	2	7	8	
	[B] A RADIO?	B. Radio	1	2	7	8	
	[C] A TELEVISION?	C. Television	1	2	7	8	
	[D] A LANDLINE PHONE?	D. Landline phone	1	2	7	8	
	[E] A REFRIGERATOR?	E. Refrigerator	1	2	7	8	
	[F] A CASSETTE PLAYER?	F. Cassette player	1	2	7	8	
	[G] A MOBILE PHONE?	G. Mobile phone	1	2	7	8	
	[H] A TABLE?	H. Table	1	2	7	8	
	[I] A CHAIR?	I. Chair	1	2	7	8	
	[J] A SOFA SET?	J. Sofa set	1	2	7	8	
	[K] A BED?	K. Bed	1	2	7	8	
	[L] A CUPBOARD?	L. Cupboard	1	2	7	8	
	[M] A CLOCK?	M. Clock	1	2	7	8	

			Yes	No	Ref	DK	
HC5	DOES ANY MEMBER OF YOUR HOUSEHOLD THAT SLEEPS HERE OWN:						
	[A] A WATCH?	A. Watch	1	2	7	8	
	[B] A BICYCLE?	B. Bicycle	1	2	7	8	
	[C] A MOTORCYCLE OR SCOOTER?	C. Motorcycle/ Scooter	1	2	7	8	
	[D] AN ANIMAL-DRAWN CART?	D. Animal drawn-cart	1	2	7	8	
	[E] A CAR OR TRUCK?	E. Car / Truck	1	2	7	8	
	[F] A BOAT WITH A MOTOR?	F. Boat with a motor	1	2	7	8	
	[G] A BOAT WITHOUT A MOTOR?	G. Boat without a motor	1	2	7	8	

SURVEY ID | | | | | | | | | | | | | | | | |

HC6	WHAT IS THE **MAIN SOURCE** OF DRINKING WATER FOR MEMBERS OF YOUR HOUSEHOLD?<br><br>*Select the main source*	Piped Water ..... 11<br>Water from borehole ..... 12<br>Water from well or spring ..... 13<br>Rain water ..... 14<br>Tanker truck ..... 15<br>Vendor ..... 16<br>Surface water (river/dam/lake/pond/stream/ canal/irrigation channel) ..... 17<br>Bottled water ..... 18<br><br>Other (specify)________ 96<br><br>Refused ..... 97<br>Don't know 98	
HC7	WHAT TYPE OF FUEL DOES YOUR HOUSEHOLD **MAINLY** USE FOR COOKING?<br><br>*Select the main source*	Electricity ..... 11<br>Gas ..... 12<br>Kerosene / Paraffin ..... 13<br>Charcoal ..... 14<br>Firewood ..... 15<br>Straw / Shrubs / Grass ..... 16<br>Animal dung ..... 17<br>No food cooked in household ..... 95<br><br>Other (specify)________ 96<br>Refused ..... 97<br>Don't know 98	
HC8	DOES ANY MEMBER OF YOUR HOUSEHOLD THAT SLEEPS HERE OWN ANY LAND THAT CAN BE USED FOR AGRICULTURE?	Yes ..... 1<br>No ..... 2<br>Refused ..... 7<br>Don't know 8	
HC9	DOES YOUR HOUSEHOLD OWN ANY LIVESTOCK, HERDS, OTHER FARM ANIMALS, OR POULTRY?	Yes ..... 1<br>No ..... 2<br>Refused ..... 7<br>Don't know 8	1 → HC10<br>2 → ME1<br>7 → ME1<br>8 → ME1

SURVEY ID | | | | | | | | | | | | | | | |

HC10	HOW MANY OF THE FOLLOWING ANIMALS DOES YOUR HOUSEHOLD HAVE?<br><br>[A] CATTLE, MILK COWS, OR BULLS?<br><br>[B] HORSES, DONKEYS, OR MULES?<br><br>[C] GOATS?<br><br>[D] SHEEP?<br><br>[E] CHICKENS?<br><br>[F] PIGS?<br>*Do not ask question if person indicated Muslim as their religion. Put 0 as the answer on your own*<br><br>*Write the number on line*<br>*Special codes:*<br>*If none, record '00'.*<br>*If 95 or more, record '95'.*<br>*If unknown, record '98'.*	Cattle, milk cows, or bulls .... ___ ___<br><br>Horses, donkeys, or mules .... ___ ___<br><br>Goats... .... ___ ___<br><br>Sheep .... ___ ___<br><br>Chickens .... ___ ___<br><br>Pigs .... ___ ___	

SURVEY ID | | | | | | | | | | | | | | | | |

MODULE F: MESSAGE EXPOSURE

SECTION I: MESSAGE EXPOSURE (ME)

NO.	QUESTION	RESPONSE CODE	SKIP
ME1	HOW OFTEN DO YOU READ A NEWSPAPER?<br><br>EVERY DAY, WEEKLY, MONTHLY, EVERY FEW MONTHS, OR NOT AT ALL?	Every day .... 1<br>Weekly .... 2<br>Monthly .... 3<br>Every few months .... 4<br>Not at all .... 5<br>Refused .... 7<br>Don't know 8	
ME2	HOW OFTEN DO YOU LISTEN TO THE RADIO?<br><br>EVERY DAY, WEEKLY, MONTHLY, EVERY FEW MONTHS, OR NOT AT ALL?	Every day .... 1<br>Weekly .... 2<br>Monthly .... 3<br>Every few months .... 4<br>Not at all .... 5<br>Refused .... 7<br>Don't know 8	1 → ME3<br>2 → ME3<br>3 → ME3<br>4 → ME3<br>5 → ME4<br>7 → ME4<br>8 → ME4
ME3	AT WHAT TIME OF THE DAY DO YOU USUALLY LISTEN TO THE RADIO??<br><br>*PROMPT EACH CHOICE. Mark "Yes" if respondent does listen to radio at the particular time and "No" if they do not.*	Early morning (before 9 am) .... A<br>Morning (between 9 am and 11 am) .... B<br>Around noon (11 am – 1 pm) .... C<br>Afternoon (1 pm – 5 pm) .... D<br>Evening (5 pm – 7 pm) .... E<br>At night (after 7 pm) .... F<br><br>Refused .... Y<br>Don't know .... Z	
ME4	HOW OFTEN DO YOU WATCH TV?<br><br>EVERY DAY, WEEKLY, MONTHLY, EVERY FEW MONTHS, OR NOT AT ALL?	Every day .... 1<br>Weekly .... 2<br>Monthly .... 3<br>Every few months .... 4<br>Not at all .... 5<br>Refused .... 7<br>Don't know 8	
ME5	HOW OFTEN DO YOU ATTEND COMMUNITY MEETINGS OR GATHERINGS (NOT INCLUDING RELIGIOUS EVENTS SUCH AS PRAYER SERVICES)?<br><br>EVERY DAY, WEEKLY, MONTHLY, EVERY FEW MONTHS, OR NOT AT ALL?	Every day .... 1<br>Weekly .... 2<br>Monthly .... 3<br>Every few months .... 4<br>Not at all .... 5<br>Refused .... 7<br>Don't know 8	

SURVEY ID | | | | | | | | | | | | | | | | |

ME6	HOW OFTEN DO YOU ATTEND GROUP RELIGIOUS EVENTS, SUCH AS PRAYER SERVICES?<br><br>EVERY DAY, WEEKLY, MONTHLY, EVERY FEW MONTHS, OR NOT AT ALL?	Every day .... 1<br>Weekly .... 2<br>Monthly .... 3<br>Every few months .... 4<br>Not at all .... 5<br>Refused .... 7<br>Don't know 8	
ME7	BEFORE TODAY, HAVE YOU RECEIVED ANY INFORMATION ABOUT ORS AND ZINC FOR THE TREATMENT OF DIARRHOEA IN THE LAST 3 MONTHS?	ORS alone .... 1<br>Zinc alone .... 2<br>Both .... 3<br>Neither .... 4<br>Refused .... 7<br>Don't know 8	1 → ME8<br>2 → ME8<br>3 → ME8<br>4 → ME10<br>7 → ME10<br>8 → ME10
ME8	FROM WHICH OF THESE SOURCES DID YOU HEAR THE MESSAGES IN THE LAST 3 MONTHS:<br><br>*Prompt each response. If "yes", select "yes". If refused to answer entire question or don't know entire question, select Refused/Don't know for all choices respectively*	Yes / No / Ref / DK	
	[A] NEWSPAPER, MAGAZINE, OR BILLBOARD?	A. Newspaper/Magazine 1 2 7 8	
	[B] RADIO?	B. Radio 1 2 7 8	
	[C] TV?	C. TV 1 2 7 8	
	[D] LOCAL COMMUNITY LEADER, SUCH AS A VILLAGE HEAD, RELIGIOUS LEADER, TEACHER OR LOCAL AUTHORITY?	D. Local leader 1 2 7 8	
	[E] SOMEONE ELSE FROM COMMUNITY SUCH AS A FAMILY MEMBER, FRIEND OR NEIGHBOUR?	E. Community member 1 2 7 8	
	[F] LOCAL HEALTH CARE WORKER AT A CLINIC, DRUG SHOP, PHARMACY OR HEALTH CENTER?	F. Health worker 1 2 7 8	
	[G] MEDICAL EDUCATOR FROM AN NGO OR NON-PROFIT ORGANIZATION?	G. Medical educator 1 2 7 8	
	[H] PERSON FROM THE DISTRICT HEALTH OFFICE TEAM OR GOVERNMENT OFFICIAL?	H. DHO 1 2 7 8	
	[X] OTHER (*specify below*)?<br><br>________________________	X. Other (specify) ________________________	

SURVEY ID | | | | | | | | | | | | | | | | |

ME9	WHAT MESSAGES DO YOU RECALL HEARING?<br><br>*Do not prompt. Mark all response mentioned as "Mentioned" and any responses not mentioned as "Not mentioned".*	Use ORS and zinc to treat diarrhoea .... A<br>ORS and zinc help children with diarrhoea recover quickly .... B<br>ORS and zinc help children with diarrhoea gain strength and energy.................... .... C<br>ORS prevents dehydration due to diarrhoea ..... D<br>ORS and zinc are the best treatments for children's diarrhoea ....E<br>Antibiotics are not recommended in most cases of diarrhea ....F<br>Antimotility (Loperamide) are never recommended for children diarrhea............. G<br>There is a Recommended Retail Price in effect for ORS and Zinc .... H<br>You should buy full treatment course of ORS and zinc at not more than 1500 UGX ....I<br><br>Other (specify)_______________ _________X<br>Refused .... Y<br>Don't know ....Z	
ME10	BEFORE TODAY, WERE YOU AWARE THAT THE GOVERNMENT OF UGANDA HAS RECOMMENDED A SPECIFIC PRICE FOR ORS AND ZINC?	Yes .... 1<br>No .... 2<br>Refused .... 7<br>Don't know 8	1 → ME11<br>2 → ME12<br>7 → ME12<br>8 → ME12
ME11	WHAT IS THE RECOMMENDED RETAIL PRICE FOR ORS AND ZINC?<br><br>*Write number on line*<br>*Special codes:*<br>*Product given for free ....9995*<br>*Refused..................................9997*<br>*Don't know............................9998*	ORS/ZINC RRP………___ , ___ ___ ___ Ugsh	
ME12	BEFORE TODAY, WERE YOU AWARE THAT THE GOVERNMENT OF UGANDA HAS RECOMMENDED A SPECIFIC PRICE FOR PAEDIATRIC AND ADULT ACTS?	Yes .... 1<br>No .... 2<br>Refused .... 7<br>Don't know 8	1 → ME13<br>2 → Mod G<br>7 → Mod G<br>8 → Mod G
ME13	WHAT IS THE RECOMMENDED RETAIL PRICE FOR ACTS?<br><br>[A] ADULT DOSE<br><br>[B] CHILD DOSE<br><br>*Write number on line*<br>*Special codes:*<br>*Product given for free ....9995*<br>*Refused..................................9997*<br>*Don't know............................9998*	Adult dose RRP ....___ , ___ ___ ___ Ugsh<br><br>Child dose RRP.............___ , ___ ___ ___ Ugsh	

SURVEY ID

MODULE G: ENDING THE SURVEY

SECTION I: SURVEY RESULT

NO.	QUESTION	RESPONSE CODE	SKIP
RESULT	*Record the result of the survey.*	Survey completed.......................... ....1<br>Household could not be found ....2<br>Household absent for an extended period ....3<br>Refused / Did not consent ....4<br>Household did not have child under 5 (0-59m)..5<br><br>Other (*specify*) _______________ _________ 6	

SECTION II: INTERVIEWER COMMENTS

NO.	QUESTION	RESPONSE CODE	SKIP
COMMENTS	*Record any comments that you have about the survey, particularly anything that may have affected the survey completion or quality of the responses.*	---<br>---<br>---<br>---<br>---<br>---	

SECTION III: GPS

NO.	QUESTION	RESPONSE CODE	SKIP
GPS	*Record the GPS location of this household using ANDROID TABLET.*<br><br>*If this does not work or is taking too long, then obtain the information using the GPS device and manually record it in the next questions*	---	
GPS_Lat	*GPS Latitude*	N or S [ ][.][ ][ ][ ][ ][ ]	
GPS_Lon	*GPS Longitude*	E [ ][ ][.][ ][ ][ ][ ][ ]	
