# Supplementary material for: Prevalence of antibiotic use for childhood diarrhoea in Uganda after an ORS scale-up intervention: a repeated cross-sectional study
Source: BMC Public Health. 2024 Aug 1;24:2084. doi: 10.1186/s12889-024-19613-4 (PMC11295441; doi:10.1186/s12889-024-19613-4)
Supplement: Supplementary file 2 — Supplementary Material 2 [file 12889_2024_19613_MOESM2_ESM.pdf]

SURVEY ID

| | | | | | | | | | | | | | | | |
|---|---|---|---|---|---|---|---|---|---|---|---|---|---|---|---|
| | | | | | | | | | | | | | | | |

# Standardized Patient Survey English Version

An assessment of the treatment of childhood illnesses in Uganda

SURVEY ID

## SECTION I: IDENTIFICATION

| NO. | QUESTION | RESPONSE CODE | SKIP |
|---|---|---|---|
| ID1 | REGION CODE<br>*In ODK, select the name* | | |
| ID2 | DISTRICT CODE<br>*In ODK, select the name* | | |
| ID3 | COUNTY CODE<br>*In ODK, select the name* | | |
| ID4 | SUB-COUNTY CODE<br>*In ODK, select the name* | | |
| ID5 | PARISH CODE<br>*In ODK, select the name* | | |
| ID6 | VILLAGE CODE<br>*In ODK, select the name* | | |
| ID7 | ENUMERATION CODE<br>*In ODK, select the name* | | |
| ID8 | SECTOR (URBAN/RURAL)<br>*In ODK, select the only option available* | Urban ........ 1<br>Rural ........ 2 | |
| ID9 | OUTLET ID<br>*Refer to Provider Listing Form.* | | |
| ID10 | OUTLET TYPE<br>*Refer to Provider Listing Form* | Drug shop........ 1<br>Pharmacy ........ 2<br>Private for-profit clinic / doctor ........ 3<br>Not-for-profit clinic ........ 4 | |
| ID11 | *Is this outlet a licensed or unlicensed outlet?* | Licensed observed........ 1<br>No license observed ........ 2 | |
| ID12 | SUPERVISOR CODE<br>*Select "Other" if you are a replacement interviewer* | | |
| ID13 | INVESTIGATOR CODE<br>*Select your name* | | |

**SURVEY ID** | | | | | | | | | | | | | |

## SECTION II: VISIT INFORMATION

| NO. | QUESTION | RESPONSE CODE | SKIP |
|---|---|---|---|
| SP1 | AT ANY POINT, DID THE PROVIDER OR RETAILER FIND OUT THAT YOU ARE PART OF THE RESEARCH STUDY? | Yes ........ 1<br>No ........ 2 | 1 → Sec VI<br>2 → SP2 |
| SP2 | FOR HOW MANY MINUTES DID YOU SPEAK WITH THE PROVIDER OR RETAILER? | Number of minutes ........ ___ ___ | |
| SP3 | DID THE PROVIDER ASK YOU ABOUT THE CHILD'S AGE? | Yes ........ 1<br>No ........ 2<br>Don't remember ........ 3 | 1 → SP5<br>2 → SP4<br>3 → SP4 |
| SP4 | DID YOU OFFER INFORMATION ABOUT THE CHILD'S AGE, EVEN THOUGH THE PROVIDER DIDN'T ASK? | Yes ........ 1<br>No ........ 2<br>Don't remember ........ 3 | |
| SP5 | DID THE PROVIDER ASK YOU WHETHER THE CHILD HAS BLOOD IN THE STOOL? | Yes ........ 1<br>No ........ 2<br>Don't remember ........ 3 | 1 → SP7<br>2 → SP6<br>3 → SP6 |
| SP6 | DID YOU OFFER INFORMATION ABOUT WHETHER THE CHILD HAS BLOOD IN THE STOOL, EVEN THOUGH THE PROVIDER DIDN'T ASK? | Yes ........ 1<br>No ........ 2<br>Don't remember ........ 3 | |
| SP7 | DID THE PROVIDER ASK YOU HOW FREQUENTLY THE CHILD WAS PASSING STOOLS? | Yes ........ 1<br>No ........ 2<br>Don't remember ........ 3 | 1 → SP9<br>2 → SP8<br>3 → SP8 |
| SP8 | DID YOU OFFER INFORMATION ON STOOL FREQUENCY TO THE PROVIDER, EVEN THOUGH THE PROVIDER DIDN'T ASK? | Yes ........ 1<br>No ........ 2<br>Don't remember ........ 3 | |
| SP9 | DID THE PROVIDER ASK YOU IF THE CHILD HAS A FEVER? | Yes ........ 1<br>No ........ 2<br>Don't remember ........ 3 | 1 → SP11<br>2 → SP10<br>3 → SP10 |
| SP10 | DID YOU OFFER INFORMATION ON WHETHER THE CHILD HAS FEVER, EVEN THOUGH THE PROVIDER DIDN'T ASK? | Yes ........ 1<br>No ........ 2<br>Don't remember ........ 3 | |

**SURVEY ID** | | | | | | | | | | | | | |

| | | | |
|---|---|---|---|
| SP11 | DID THE PROVIDER ASK YOU IF THE CHILD IS VOMITING? | Yes ........ 1<br>No ........ 2<br>Don't remember ........ 3 | 1 → SP13<br>2 → SP12<br>3 → SP12 |
| SP12 | DID YOU OFFER INFORMATION ABOUT WHETHER THE CHILD WAS VOMITING, EVEN THOUGH THE PROVIDER DIDN'T ASK? | Yes ........ 1<br>No ........ 2<br>Don't remember ........ 3 | |
| SP13 | DID THE PROVIDER ASK YOU ABOUT ANY SIGNS OF DEHYDRATION, SUCH AS WHETHER THE CHILD HAD SUNKEN EYES OR IF THE SKIN LOOKED DRY? | Yes ........ 1<br>No ........ 2<br>Don't remember ........ 3 | 1 → SP15<br>2 → SP14<br>3 → SP14 |
| SP14 | DID YOU OFFER INFORMATION ABOUT WHETHER THE CHILD WAS DEHYDRATED, EVEN THOUGH THE PROVIDER DIDN'T ASK? | Yes ........ 1<br>No ........ 2<br>Don't remember ........ 3 | |
| SP15 | DID THE PROVIDER ASK YOU WHETHER YOU GAVE THE CHILD ANY MEDICINES? | Yes ........ 1<br>No ........ 2<br>Don't remember ........ 3 | 1 → SP17<br>2 → SP16<br>3 → SP16 |
| SP16 | DID YOU OFFER INFORMATION ABOUT WHETHER YOU GAVE THE CHILD ANY MEDICINE, EVEN THOUGH THE PROVIDER DIDN'T ASK? | Yes ........ 1<br>No ........ 2<br>Don't remember ........ 3 | |
| SP17 | DID THE PROVIDER ASK YOU ANY QUESTIONS ABOUT THE CHILD'S URINE, SUCH AS WHETHER THE CHILD WAS URINATING NORMALLY OR HAD DARKER URINE? | Yes ........ 1<br>No ........ 2<br>Don't remember ........ 3 | 1 → SP19<br>2 → SP18<br>3 → SP18 |
| SP18 | DID YOU OFFER INFORMATION ABOUT THE CHILD'S URINE, EVEN THOUGH THE PROVIDER DIDN'T ASK? | Yes ........ 1<br>No ........ 2<br>Don't remember ........ 3 | |
| SP19 | DID THE PROVIDER ASK ABOUT THE CHILD'S GENERAL BEHAVIOUR, SUCH AS WHETHER THE CHILD WAS ACTIVE AND PLAYFUL OR TIRED WITH NO ENERGY? | Yes ........ 1<br>No ........ 2<br>Don't remember ........ 3 | 1 → SP21<br>2 → SP20<br>3 → SP20 |
| SP20 | DID YOU OFFER INFORMATION ABOUT THE CHILD'S GENERAL BEHAVIOUR, EVEN THOUGH THE PROVIDER DIDN'T ASK? | Yes ........ 1<br>No ........ 2<br>Don't remember ........ 3 | |

**SURVEY ID** | | | | | | | | | | | | | |

| | | | |
|---|---|---|---|
| SP21 | DID THE PROVIDER ASK ABOUT WHETHER THE CHILD WAS HAVING ANY PAIN IN THE STOMACH AREA? | Yes .......... 1<br>No .......... 2<br>Don't remember .......... 3 | 1 → SP23<br>2 → SP22<br>3 → SP22 |
| SP22 | DID YOU OFFER INFORMATION ABOUT WHETHER THE CHILD WAS HAVING PAIN IN STOMACH AREA, EVEN THOUGH THE PROVIDER DIDN'T ASK? | Yes .......... 1<br>No .......... 2<br>Don't remember .......... 3 | |
| SP23 | DID THE PROVIDER ASK YOU WHETHER YOU ARE GIVING ANY FLUIDS TO THE CHILD? | Yes .......... 1<br>No .......... 2<br>Don't remember .......... 3 | 1 → SP25<br>2 → SP24<br>3 → SP24 |
| SP24 | DID YOU OFFER INFORMATION ABOUT WHETHER YOU ARE GIVING ANY FLUIDS TO THE CHILD, EVEN THOUGH THE PROVIDER DIDN'T ASK? | Yes .......... 1<br>No .......... 2<br>Don't remember .......... 3 | |
| SP25 | DID THE PROVIDER ASK ABOUT WHAT THE CHILD HAS EATEN? | Yes .......... 1<br>No .......... 2<br>Don't remember .......... 3 | 1 → SP27<br>2 → SP26<br>3 → SP26 |
| SP26 | DID YOU OFFER INFORMATION ABOUT WHAT THE CHILD HAS EATEN, EVEN THOUGH THE PROVIDER DIDN'T ASK? | Yes .......... 1<br>No .......... 2<br>Don't remember .......... 3 | |
| SP27 | DID THE PROVIDER ASK YOU HOW MUCH MONEY YOU HAVE? | Yes .......... 1<br>No .......... 2<br>Don't remember .......... 3 | 1 → SP29<br>2 → SP28<br>3 → SP28 |
| SP28 | DID YOU OFFER INFORMATION ABOUT HOW MUCH MONEY YOU HAVE, EVEN THOUGH THE PROVIDER DIDN'T ASK? | Yes .......... 1<br>No .......... 2<br>Don't remember .......... 3 | |
| SP29 | DID THE PROVIDER ASK YOU ANY OTHER QUESTIONS THAT WERE NOT ALREADY MENTIONED? | Yes .......... 1<br>No .......... 2<br>Don't remember .......... 3 | 1 → SP30<br>2 → SP31<br>3 → SP31 |
| SP30 | WHAT OTHER QUESTIONS DID THE PROVIDER ASK YOU? | ---------------------------------------- | |
| SP31 | DID YOU TELL THE PROVIDER ANYTHING ELSE ABOUT THE CHILD OR THE ILLNESS THAT WAS NOT ALREADY MENTIONED? | Yes .......... 1<br>No .......... 2<br>Don't remember .......... 3 | 1 → SP32<br>2 → DG1<br>3 → DG1 |
| SP32 | WHAT ELSE DID YOU TELL THE PROVIDER? | ---------------------------------------- | |

**SURVEY ID** | | | | | | | | | | | | | |

## SECTION III: DIAGNOSIS

| NO. | QUESTION | RESPONSE CODE | SKIP |
|---|---|---|---|
| DG1 | DID THE PROVIDER TELL YOU WHAT ILLNESS THE CHILD IS SUFFERING FROM?<br><br>*(I.e. Did the provider say, :I think this is ________(Diarrhea, Malaria, Pneumonia, etc.) or "It sounds like your child has ________" as opposed to just giving treatment without discussing the diagnosis first.)* | Yes .......... 1<br>No .......... 2<br>Don't remember .......... 3 | 1 → DG2<br>2 → PR1<br>3 → PR1 |
| DG2 | WHAT ILLNESS DID THE PROVIDER SAY YOUR CHILD IS SUFFERING FROM? | ---------------------------------------------------------- | |

## SECTION IV: PRESCRIPTIONS AND TREATMENTS

| NO. | QUESTION | RESPONSE CODE | SKIP |
|---|---|---|---|
| PR1 | DID YOU RECEIVE EITHER MEDICINES OR A WRITTEN PRESCRIPTION SLIP FROM THE OUTLET? | Purchased medicines .......... 1<br>Received prescription slip .......... 2<br>Both .......... 3<br>Neither medicines or prescription slip .......... 4 | 1 → PR3<br>2 → PR3<br>3 → PR3<br>4 → PR2 |
| PR2 | WHY DIDN'T YOU RECEIVE ANY MEDICINES OR A PRESCRIPTION SLIP?<br><br>*Do not prompt. Mark all response mentioned as "Mentioned" and any responses not mentioned as "Not mentioned".* | The provider requested to see the child .......... A<br><br>The provider didn't have any medicines for diarrhoea in stock .......... B<br><br>The provider recommended home-made remedies or fluids .......... C<br><br>Other reasons (specify) .......... X<br><br>[________________________________]<br><br>Don't remember .......... Z | All responses → Sec VI |
| **INSTRUCTIONS:** *Collect all medicines and/or prescriptions from the standardized patient and complete the following questions.* | | | |
| PR3 | HOW MANY DIFFERENT MEDICINES DID THE PROVIDER DISPENSE OR PRESCRIBE? | Number of medicines .......... ___ ___ | |

**SURVEY ID** | | | | | | | | | | | | | |

| | | | |
|---|---|---|---|
| PR4 | WHAT ARE THE NAMES OF THE MEDICINES DISPENSED OR PRESCRIBED?<br><br>*Record the names of ALL medicines prescribed or dispensed. If the medicine is unmarked or not distinguishable, record a description of the medicine, such as "Red and yellow capsule." If you are unsure, do not try to guess.* | Medicine 1 ____________________ A<br>Medicine 2 ____________________ B<br>Medicine 3 ____________________ C<br>Medicine 4 ____________________ D<br>Medicine 5 ____________________ E<br>Medicine 6 ____________________ F<br>Medicine 7 ____________________ G<br>Medicine 8 ____________________ H | |
| PR5 | CODE THE MEDICINE NAMES INTO CATEGORIES.<br><br>*Using the treatment catalogue in your field manual, please categorize the medicines mentioned above* | Medicine 1 .................... ___ ___<br>Medicine 2 .................... ___ ___<br>Medicine 3 .................... ___ ___<br>Medicine 4 .................... ___ ___<br>Medicine 5 .................... ___ ___<br>Medicine 6 .................... ___ ___<br>Medicine 7 .................... ___ ___<br>Medicine 8 .................... ___ ___<br><br>Co-pack of ORS and Zinc .................... 1<br>Oral rehydration salts (ORS) .................... 2<br>Zinc (tablets or syrup) .................... 3<br>Amoxicillin (tablets or syrup) .................... 4<br>Cotrimoxazole (tablets or syrup) .................... 5<br>Metronidazole (tablets or syrup) .................... 6<br>Other antibiotics (tablets or syrup) .................... 7<br>Anti-motility .................... 8<br>Artemisinin combination therapy (ACTs) .................... 9<br>Other antimalarials – not ACTs .................... 10<br>Paracetamol .................... 11<br>IV fluids .................... 12<br>Injection (antibiotic or non-antibiotic) .................... 13<br>Home remedy (coconut water, juice, etc.) .................... 14<br>Herbs .................... 15<br>Other .................... 96<br>Refused .................... 97<br>Don't know .................... 98 | |
| PR6 | WAS ORS DISPENSED OR PRESCRIBED BY THE PROVIDER? | Yes .................... 1<br>No .................... 2 | 1 → PR7<br>2 → PR12 |

**SURVEY ID** | | | | | | | | | | | | | |

| | | | |
|---|---|---|---|
| PR7 | HOW MANY SACHETS OF ORS DID THE PROVIDER DISPENSE?<br><br>*Write the number on line.*<br>*Special code:*<br>*If ORS was not given……..95* | Number of ORS sachets......................... ___ ___ | |
| PR8 | DID THE PROVIDER GIVE YOU ANY INFORMATION ABOUT HOW TO PREPARE AND GIVE ORS TO YOUR CHILD? | Yes .......... 1<br>No .......... 2<br>Don't remember .......... 3 | 1 → PR9<br>2 → PR12<br>3 → PR12 |
| PR9 | DID THE PROVIDER TELL YOU TO USE DRINKING WATER WHEN PREPARING ORS? | Yes .......... 1<br>No .......... 2<br>Don't remember .......... 3 | |
| PR10 | HOW OFTEN DID THE PROVIDER TELL YOU TO GIVE ORS TO THE CHILD? | Once per day .......... 11<br>Twice per day.......... 12<br>Three times per day.......... 13<br>Four times or more per day .......... 14<br>After each time the child passes stool .......... 15<br>No instruction on frequency given .......... 16<br><br>Other (specify) ________________________ 96<br><br>Don't remember .......... 98 | |
| PR11 | FOR HOW LONG DID THE PROVIDER TELL YOU TO GIVE ORS TO THE CHILD?<br><br>*Write the number on line.*<br>*Special code:*<br>*Until the illness stopped ............. 80*<br>*No instructions on how long to offer ORS to child was given .................. 95*<br>*Other (specify)............................ 96*<br>*Don't remember ......................... 98* | Number of days...................................... ___ ___ | |
| PR12 | WERE ZINC TABLETS OR SYRUP DISPENSED OR PRESCRIBED BY THE PROVIDER? | Yes .......... 1<br>No .......... 2 | 1 → PR13<br>2 → FF1 |
| PR13 | HOW MANY OF ZINC TABLETS/ SYRUPS WERE DISPENSED OR PRESCRIBED BY THE PROVIDER?<br><br>*Write the number on line.*<br>*Special code:*<br>*If zinc tablets/syrup not given……..95* | Number of zinc tablets/syrups................ ___ ___ | |

**SURVEY ID** | | | | | | | | | | | | | |

| | | | |
|---|---|---|---|
| PR14 | DID THE PROVIDER GIVE DOSING OR PREPARATION INSTRUCTIONS FOR GIVING ZINC TO YOUR CHILD? | Yes .......... 1<br>No .......... 2<br>Don't remember .......... 8 | 1 → PR15<br>2 → FF1<br>3 → FF1 |
| PR15 | HOW MANY ZINC TABLETS OR SYRUP CUPS DID THE PROVIDER INSTRUCT TO GIVE TO YOUR CHILD? | Half tablet/syrup cup .......... 1<br>One tablet/syrup cup .......... 2<br>Two tablets/syrup cups .......... 3<br>Three tablets/syrup cups or more .......... 4<br>No instruction on dosing was given .......... 5<br><br>Other (*specify*) ________________ 6<br><br>Don't remember .......... 8 | |
| PR16 | HOW OFTEN DID THE PROVIDER INSTRUCT TO GIVE ZINC TO YOUR CHILD? | Once per day .......... 11<br>Twice per day .......... 12<br>Three times per day .......... 13<br>Four times or more per day .......... 14<br>After each time the child passes stool .......... 15<br>No instruction on frequency given .......... 16<br><br>Other (specify) ________________ 96<br><br>Don't remember .......... 98 | |
| PR17 | FOR HOW LONG DID THE PROVIDER TELL YOU TO GIVE ZINC TO THE CHILD?<br><br>*Write the number on line.*<br>*Special code:*<br>*Until the illness stopped .......... 80*<br>*No instructions on how long to offer zinc to child was given .......... 95*<br>*Other (specify) .......... 96*<br>*Don't remember .......... 98* | Number of days .......... ___ ___ | |

**SURVEY ID** | | | | | | | | | | | | | |

## SECTION V: FEES AND FOLLOW-UP

| NO. | QUESTION | RESPONSE CODE | SKIP |
|---|---|---|---|
| FF1 | DID THE PROVIDER CHARGE YOU ANY MONEY? | Yes ........ 1<br>No ........ 2 | 1 → FF2<br>2 → Sec VI |
| FF2 | HOW MUCH DID YOU PAY FOR:<br>[A] CONSULTATION?<br>[B] MEDICINES?<br>[C] TOTAL?<br>*Write '000' if cost of any component was free*<br>*Write '998' if cost of any component or the total is unknown* | A. Consultation ........___, ___ ___ ___ UGX<br>B. Medicines ........___, ___ ___ ___ UGX<br>C. Total ........___, ___ ___ ___ UGX | |
| FF3 | DID THE PROVIDER INSTRUCT YOU TO RETURN OR TO TAKE YOUR CHILD TO A HEALTH FACILITY FOR ANY REASON? | Yes ........ 1<br>No ........ 2<br>Don't remember ........ 3 | 1 → FF4<br>2 → Sec VI<br>3 → Sec VI |
| FF4 | WHAT REASONS DID THE PROVIDER GIVE FOR RETURNING OR TAKING YOUR CHILD TO A HEALTH FACILITY?<br>*Do not prompt. Mark all response mentioned as "Mentioned" and any responses not mentioned as "Not mentioned* | Repeated vomiting ........ A<br>Fever ........ B<br>Weakness ........ C<br>Profuse diarrhoea ........ D<br>Eating or drinking less than normal ........ E<br>Blood in stool ........ F<br>Marked thirst ........ G<br>Other (specify) ________________ X | |

## SECTION VI: STANDARDIZED PATIENT BACKGROUND

| NO. | QUESTION | RESPONSE CODE | SKIP |
|---|---|---|---|
| BA1 | WHAT IS YOUR NAME? | ------------------------------------------------ | |
| BA2 | WHAT IS YOUR AGE?<br>*Record the age on the response line.*<br>*Special codes:*<br>*97 = Refused*<br>*98 = Don't know* | Age of standardized patient ........ ___ ___ | |

**SURVEY ID** | | | | | | | | | | | | | |

| | | | |
|---|---|---|---|
| BA3 | WHAT IS YOUR PRIMARY OCCUPATION? | Housewife ........ 11<br>Farmer ........ 12<br>Shopkeeper ........ 13<br>Teacher, police, public servant ........ 14<br>Office or company worker ........ 15<br>Doctor, lawyer, etc. ........ 16<br><br>Other (*specify*) ________ 96<br>Refused ........ 97 | |
| BA4 | WHAT IS YOUR MARITAL STATUS? | Currently married ........ 1<br>Never been married ........ 2<br><br>Other (*specify*) ________ 6<br>Refused ........ 7 | |
| BA5 | WHAT IS YOUR TRIBE? | Muganda ........ 1<br>Munyankole ........ 2<br>Musoga ........ 3<br>Mukiga ........ 4<br>Ateso ........ 5<br><br>Other (*specify*) ________ 6<br>Refused ........ 7 | |
| BA6 | WHAT LANGUAGE DID YOU USE WHEN SPEAKING WITH THE HEALTHCARE PROVIDER? | Ateso ........ 11<br>English ........ 12<br>Luganda ........ 13<br>Lugbara ........ 14<br>Luo ........ 15<br>Runyankole ........ 16<br>Rutooro ........ 17<br><br>Other (*specify*) ________ 96 | |
| BA7 | HOW MANY CHILDREN UNDER THE AGE OF 5 (0-59 MONTHS) DO YOU HAVE?<br><br>*Write number on the line.*<br>*Special codes:*<br>*97 = Refused*<br>*98 = Don't know* | Number of children ........ ___ ___ | |
| BA8 | WHAT IS THE AGE OF YOUR YOUNGEST CHILD **IN MONTHS**?<br><br>*Write number on the line.*<br>*Special codes:*<br>*97 = Refused*<br>*98 = Don't know* | Age of youngest child ........ ___ ___ | |
| BA9 | WHAT IS THE GENDER OF YOUR YOUNGEST CHILD? | Male ........ 1<br>Female ........ 2<br>Refused ........ 7 | |

**SURVEY ID** | | | | | | | | | | | | | |

| | | | |
|---|---|---|---|
| BA10 | WHAT IS YOUR CONTACT NUMBER?<br><br>*If refused, write 9998* | ------------------------------------------------------------ | |

## SECTION VII: SURVEY RESULT

| NO. | QUESTION | RESPONSE CODE | SKIP |
|---|---|---|---|
| RESULT | *Record the result of the survey.* | I purchased medicines from the outlet ............... 1<br>I received a prescription from the outlet ............ 2<br>Outlet did not believe I needed medicines ......... 3<br>Outlet referred me elsewhere for treatment........ 4<br>Outlet found out I was conducting SP interview 5<br><br>Other (*specify*) ___________________________<br>_____________________________________ 6 | |
| COMMENTS | *Record any comments that you have about the survey, particularly anything that may have affected the survey completion or quality of the responses.* | --------------------------------------------------------<br>--------------------------------------------------------<br>--------------------------------------------------------<br>--------------------------------------------------------<br>--------------------------------------------------------<br>-------------------------------------------------------- | |

## SECTION VIII: GPS

| NO. | QUESTION | RESPONSE CODE | SKIP |
|---|---|---|---|
| GPS | *Record the GPS location of this retailer or health care provider using ANDROID TABLET.*<br><br>*If this does not work or is taking too long, then obtain the information using the GPS device and manually record it in the next questions* | -------------------------------------------------------- | |
| GPS_Lat | *GPS Latitude* | N or S [ ] [ ] . [ ] [ ] [ ] [ ] [ ] | |
| GPS_Lon | *GPS Longitude* | E [ ] [ ] [ ] . [ ] [ ] [ ] [ ] [ ] | |
